# Effects of exercise on cognitive function in patients with depression: a three-level meta-analysis with dose-response and exploratory mediation analyses

**DOI:** 10.1186/s12966-026-01902-3

**Published:** 2026-03-11

**Authors:** Cong Liu, Rao Chen, Shuqi Jia, Zhaohui Guo, Chen Wei, Xing Wang

**Affiliations:** 1 School of Physical Education, Shanghai University of Sport, Shanghai, 200438 China; 2 Shanghai I and C Foreign Languages School, Shanghai, 201399 China; 3 College of Sports Science, Shenyang Normal University, Shenyang, 110034 China

**Keywords:** Exercise, Depression, Cognitive function, Meta-analysis

## Abstract

**Background:**

Cognitive impairment is common among individuals with depression and contributes to functional impairment and poor treatment outcomes. Exercise, as a non-pharmacological intervention, has been increasingly recognized as a promising approach for improving cognitive function. However, the dose-response relationship underlying these effects remains poorly understood, and the mechanisms through which exercise may influence cognition or depressive symptoms have yet to be clarified. To address these gaps, this study used a three-level meta-analysis to quantify the effects of exercise on cognitive function in depression, examine potential moderators and dose-response patterns, and conduct an exploratory mediation analysis testing whether cognitive changes mediate exercise-related improvements in depressive symptoms.

**Methods:**

We searched China National Knowledge Infrastructure (CNKI), Wanfang Data, Web of Science, Embase, the Cochrane Library, and PubMed for experimental studies evaluating the effects of exercise interventions on cognitive function in patients with depression, from database inception to May 30, 2025. Risk of bias was assessed using the Cochrane Risk of Bias 2 (RoB 2) tool, and certainty of evidence was graded with GRADEpro. Three-level meta-analyses were conducted with the metafor package in R.

**Results:**

Thirty-one studies, including 2,324 participants with depression, were included. Exercise type included aerobic, resistance/strength training, mind-body practices, and multicomponent exercises. Exercise frequency ranged from 1 to 5 sessions per week; intervention duration from 3 to 16 weeks; and exercise intensity was categorized as low, moderate, or moderate to high. Session duration ranged from 30 to 180 min. A three-level meta-analysis showed that exercise improved cognitive function (Hedges’ g (g) = 0.24; 95% confidence Interval (CI), 0.14 to 0.33; *p* < 0.001). Moderator analyses indicated significant influences of exercise intensity, inpatient status, intervention content, study design, and weekly exercise time (all *p* < 0.05). Moderate-intensity interventions produced the largest gains (g = 0.36; 95% CI, 0.23 to 0.50; *p* < 0.05). Dose-response modelling suggested that weekly exercise volumes below approximately 176 min may yield limited benefit (prediction interval crossing zero). In an exploratory mediation analysis, the indirect effect of exercise on depressive symptoms via cognition was insignificant (a×b = 0.02; 95% CI, -0.00 to 0.04), corresponding to a proportion mediated of 5.3%.

**Conclusions:**

Exercise is associated with improvements in cognitive function among individuals with depression. Rather than indicating a universal recommended dose, the dose–response analysis revealed a minimum weekly activity threshold, with cognitive benefits becoming more likely when moderate-intensity exercise exceeds approximately 176 min per week. Current evidence does not support cognitive improvement as the primary mediating pathway through which exercise influences depressive symptoms, and this finding should be interpreted cautiously. Further multicenter randomized controlled trials with rigorous methodology and standardized cognitive assessments are needed to refine modality-specific dose-response patterns and clarify potential mediating pathways.

**Trial registration:**

PROSPERO registration number CRD42025645011.

**Supplementary Information:**

The online version contains supplementary material available at 10.1186/s12966-026-01902-3.

## Introduction

Depression is a mood disorder characterized by a persistent and marked lowering of mood, with high lifetime prevalence and a high risk of recurrence [1]. According to the World Health Organization (WHO), an estimated 280 million people worldwide are affected by depression [2]. The lifetime prevalence is 6.8%, and the lifetime disability rate has been reported to be as high as 47% [3, 4]. By 2030, depression is projected to become the leading contributor to the global burden of disease [5]. Beyond its personal and familial impact, depression also carries a substantial economic burden: in the United States, the financial cost among adults increased from USD 236.6 billion in 2010 to USD 326.2 billion in 2018 [6].

Patients with depression frequently exhibit cognitive deficits, now recognized as a core diagnostic feature of the disorder [7, 8]. Executive dysfunction is reflected in poorer performance on tasks requiring planning, decision-making, set-shifting, and the organization and reasoning of information [9]; attentional problems include difficulty concentrating and sustaining attention; memory problems include impaired short-term memory and marked forgetfulness [9]; and slowed information-processing presents as psychomotor slowing and a subjective sense of “cognitive fog” [10]. Consistent with these observations, systematic reviews and meta-analyses show that, compared with healthy controls, individuals in a depressive state display deficits in executive function, attention, and memory [11]. Cognitive impairment spans the course of illness - emerging in the prodromal phase, persisting during acute episodes, and often remaining in remission [12, 13]. These deficits reduce the likelihood of antidepressant response and remission and increase the risk of relapse [14]. Because treatment goals include clinical remission, restoration of functioning, and improved quality of life, comprehensive care must address affective, somatic, and cognitive symptoms [15].

As a potential non-pharmacological treatment, exercise may improve cognitive function [16] and alleviate mood symptoms [17]. Accordingly, multiple national guidelines-and the WHO-recommend incorporating exercise into depression care pathways [18, 19]. Nevertheless, clinical findings remain mixed. Hoffman et al. reported that a 16-week aerobic-exercise program improved executive function in patients with depression, with only a weak association between reductions in depressive-symptom severity and cognitive gains [20]. In contrast, Lavretsky et al. found that a 12-week tai chi intervention did not improve cognitive function, and changes in cognition were unrelated to depressive-symptom severity [21].

Despite inconsistencies in clinical findings, a substantial body of basic science and neuroimaging research provides mechanistic support for the cognitive benefits of exercise in individuals with depression. Cognitive impairments in depression are closely linked to dysfunction across several large-scale brain networks, including hyperactivation of the default mode network at rest, reduced efficiency of the executive control network, and decreased prefrontal cortical activity-all of which are associated with deficits in inhibitory control and self-referential processing [22, 23]. In addition, depressed individuals exhibit biological dysregulation, such as reduced levels of brain-derived neurotrophic factor and diminished neural plasticity [24–26]. Regular physical exercise has been shown to enhance the functional efficiency and connectivity of higher-order cognitive networks, including the default mode and prefrontal executive networks, thereby providing a neural basis for cognitive improvement [27]. Exercise also increases peripheral and central brain-derived neurotrophic factor levels and promotes synaptic plasticity [28]. Accumulating evidence demonstrates that exercise can significantly increase hippocampal volume and improve memory and other cognitive functions [29]. Taken together, these neurobiological findings suggest that exercise has plausible mechanisms through which it could improve cognition in depression, even if clinical results remain variable.

Despite this plausible mechanistic foundation, meta-analyses and systematic reviews evaluating the cognitive effects of exercise in depression have reported inconsistent results. Ren et al. reported that exercise improved overall executive function [30] and global cognition [31], with notable gains in attention, memory, and processing speed [31]. In contrast, Guo et al. found benefits limited to attention, with no significant effects on executive function, memory, or processing speed [32]. Brondino et al. reported no significant improvement in either global cognition or cognitive subdomains [33]. These discrepancies may be attributable to differences in the number of included studies and the meta-analytic methods employed.

Building on prior work, notable heterogeneity remains in the reported cognitive benefits of exercise for individuals with depression. Although several reviews have attempted to examine potential moderators, their findings have been inconsistent, and the dose-response relationship between exercise and cognitive improvement has yet to be clearly established. Moreover, some previous reviews restricted inclusion to English-language publications, raising the risk of language-related publication bias. The potential mediating role of cognitive function in the relationship between exercise and depressive-symptom improvement has also not been systematically tested, limiting understanding of the broader mechanistic pathways through which exercise may exert its therapeutic effects.

From a methodological perspective, although three-level meta-analytic models have been used to evaluate exercise interventions, their application within the field of cognitive outcomes in depression remains limited. Existing dose-response analyses have focused primarily on linear associations involving total intervention time. For example, Ren and colleagues assessed the linear relationship between total exercise duration and cognitive improvement within a three-level framework but reported no significant effects [31]. In contrast, weekly exercise duration, which aligns more closely with how exercise prescriptions are structured in clinical practice, has not been incorporated into continuous dose-response analyses. International guidelines-including those issued by the WHO and the American College of Sports Medicine (ACSM)-identify weekly activity volume (e.g., 150–300 min of moderate-intensity activity) as the primary dosing metric [34, 35], and depression-related exercise studies similarly operationalize dose in minutes per week [36]. Nevertheless, the literature lacks systematic modeling of both linear and nonlinear weekly dose-response curves, leaving the pattern of association between exercise dose and cognitive gains unclear. Furthermore, whether cognitive function mediates the effects of exercise on depressive symptoms has yet to be examined within an integrated analytic framework.

The present study addresses these gaps through several methodological and theoretical extensions. First, by applying a three-level random-effects model, we integrated multiple effect sizes within individual studies, yielding more stable and precise estimates. Second, building upon prior work that examined only total exercise duration, we conducted the first systematic dose-response analysis based on weekly exercise duration, modeling both linear and nonlinear associations to provide evidence that is more directly applicable to clinical practice. Third, we performed an exploratory mediation analysis to evaluate whether cognitive function serves as a potential mechanism through which exercise influences depressive symptoms. Additionally, by expanding our search to include both English- and Chinese-language databases, we reduced the risk of language-related bias. Collectively, these advances in dose specification, methodological integration, and mechanistic exploration offer substantive contributions to the existing literature and provide more precise evidence to inform clinically actionable exercise prescriptions for individuals with depression.

## Methods

This systematic review and meta-analysis were conducted in accordance with the Cochrane Handbook for Systematic Reviews of Interventions and reported in line with the PRISMA 2020 guidelines [37]. The protocol was registered with PROSPERO (CRD42025645011). In keeping with open-science practices, we have deposited all analysis code and de-identified data on the Open Science Framework (OSF): https://osf.io/62jkt/?view_only=749bcc63321c46a9a2fa33f3401b568b.

### Eligibility criteria

Following the Population (P), Intervention (I), Comparison (C), Outcome (O), and Study design (S) framework:

P: Patients diagnosed with depression according to the International Classification of Diseases (ICD), the Diagnostic and Statistical Manual of Mental Disorders (DSM), or other validated diagnostic tools; no age restrictions. Exclusions: dementia, major neurological disorders, current substance dependence, or psychotic disorders.

I: Exercise was defined as structured, repeated exercise training rather than acute (single-session) exercise [38]. Interventions were required to include multiple exercise sessions delivered over a defined training period, regardless of total duration. The experimental arm included studies in which participants received exercise alone, exercise combined with other treatments, or exercise added to standard care. Exercise type was unrestricted (aerobic, resistance, mind-body, or multicomponent).

C: Pharmacotherapy, treatment as usual, stretching/relaxation, occupational therapy, or other non-exercise controls. Trials were excluded if the control arm added components likely to directly improve cognition (e.g., cognitive training) that were not matched between groups.

O: Global cognition or at least one cognitive domain, assessed with validated measures such as the Trail Making Test (TMT), Digit Symbol Substitution Test (DSST), Digit Span Test (DST), Controlled Oral Word Association Test (COWAT), Go/No-Go, or Stroop tasks.

S: Randomized controlled trials (RCTs) or quasi-experimental studies with a parallel control group.

#### Exclusion criteria

Non-Chinese/non-English publications; cognitive outcome data unobtainable after contacting authors; interventions not meeting the definition of exercise (e.g., single-bout exercise); reviews or conference abstracts; and studies in which the effect of exercise could not be isolated because of unmatched co-interventions (e.g., cognitive training added only to the experimental arm).

### Data sources and search strategy

We systematically searched six electronic databases (China National Knowledge Infrastructure (CNKI), Wanfang Data, PubMed, Embase, the Cochrane Library, and Web of Science(WOS)) for experimental studies evaluating the effects of exercise interventions on cognitive function in patients with depression. The search covered each database from inception to 30 January 2025 and was updated on 30 May 2025. Search terms included “exercise”, “aerobic exercise”, “tai chi”, “qigong”, “physical activity”, “depressive disorder”, “major depressive disorder”, “depression”, “cognitive function”, and “executive function”. We combined controlled vocabulary (MeSH and Emtree) with free text keywords using the Boolean operators OR and AND. Detailed search strings for each database are provided in Table 1. We also screened the reference lists of relevant systematic reviews and meta analyses to identify additional studies. Studies identified from prior reviews were treated as records identified from other sources and were subjected to the same deduplication, screening, and eligibility assessment procedures as database-derived records.

### Selection process

**Fig. 1:**
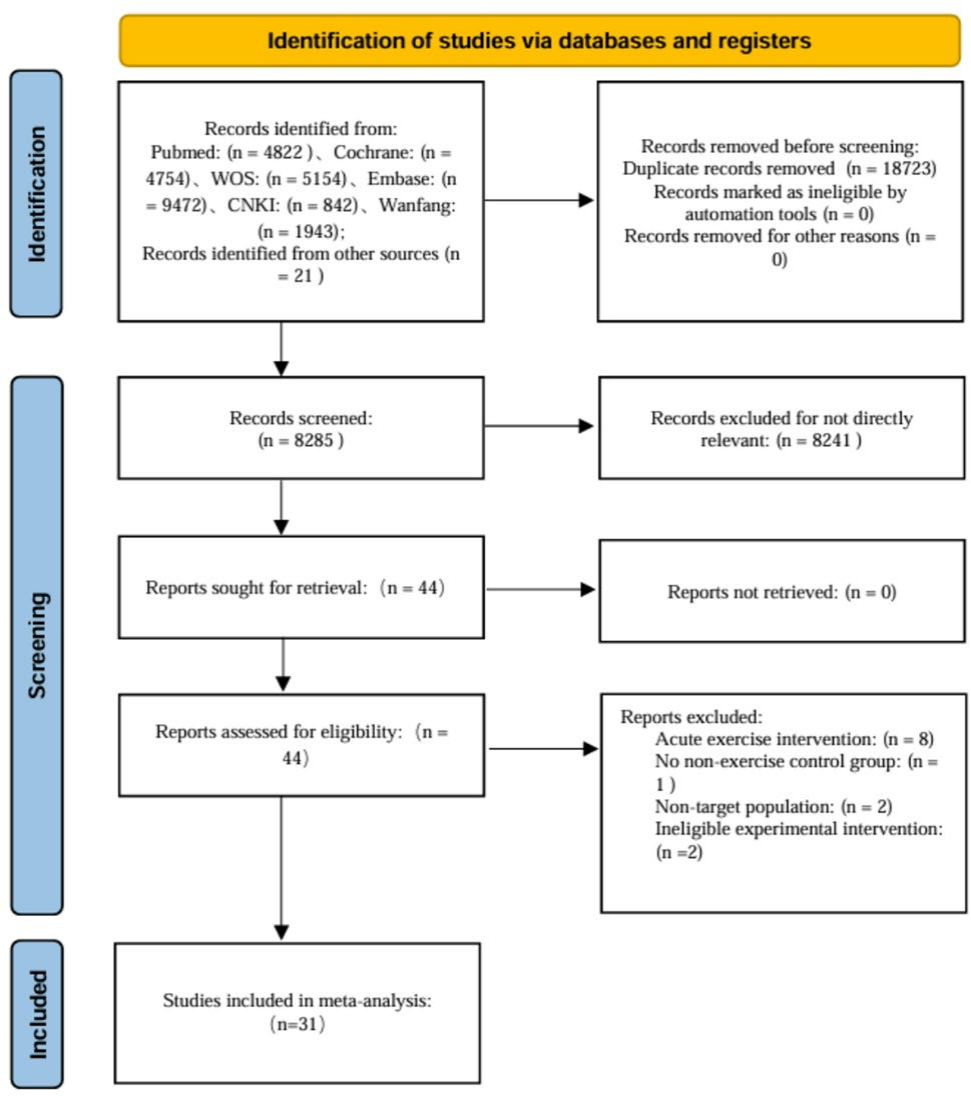
PRISMA 2020 flow diagram of study selection

Two reviewers (CW and RC) independently imported all records into EndNote X9 and removed duplicates. They screened titles and abstracts to exclude irrelevant studies, then retrieved and reviewed the full texts of potentially eligible articles against the prespecified criteria. Any disagreements were resolved through discussion; if disagreement persisted, a third reviewer (XW) adjudicated until consensus was reached. A PRISMA flow diagram summarizes the selection process (Fig. 1).

### Data extraction and coding

Two reviewers (SQ J and ZH G) independently extracted data using a piloted Microsoft Excel form. Extracted information included study characteristics (first author, year, country, sample size, mean age, sex, inpatient status, study design, and depression score), intervention characteristics (exercise type, session duration, weekly frequency, intensity, and cycle), and cognitive outcomes. In addition to collecting basic study characteristics, we also extracted change scores for each cognitive outcome in both the experimental and control groups, calculated as the post-intervention value minus the baseline value (post - pre). When a study included two or more eligible experimental or control groups, all qualifying groups were extracted separately and entered into the analysis. If required means or standard deviations were not reported in the original publication, we contacted the corresponding authors to obtain supplementary data. For continuous outcomes, the mean change was calculated as: Mean_change_ = Mean_post_-Mean_baseline_. If the standard deviation of the change score (SD_change_) was not reported, it was estimated according to the recommendations of the Cochrane Handbook using baseline and post-intervention standard deviations: SD_change_=$S{D}_{change}=\sqrt{S{D}_{baseline}^{2}+S{D}_{post}^{2}-2\times{r}\times{SD}_{baseline}\times{SD}_{post}}$, where r represents the correlation coefficient between baseline and post-intervention measurements. Because this coefficient was not reported in most included studies, we followed the approach commonly used in exercise-intervention meta-analyses and assumed *r* = 0.50.

Because this review examined moderators, we coded variables as follows. Cognitive outcomes were grouped into domains: executive function, memory, attention, processing speed, and verbal ability. Exercise type was categorized as aerobic, resistance, mind–body, or multicomponent [31]. Exercise frequency was categorized as 1 to 2 or 3 to 5 sessions per week. Session duration was categorized as 30 to 60 min or greater than 60 min [31]. Exercise intensity was categorized as low, moderate, or moderate to high [16]. Exercise cycle was categorized as 3 to 6 weeks, 8 to 12 weeks, or greater than 12 weeks. Age was categorized as adolescents, young adults, middle-aged adults, and older adults [30]. Study design was coded as RCTs or nonRCTs. The experimental content was coded as exercise only or exercise plus other therapy. The control condition was coded as active control or passive control. Inpatient status was coded as inpatient or noninpatient. Additional coding details are provided in Table 2.

In coding exercise intensity, the present study did not rely solely on the intensity labels reported in the original trials. Instead, we referred to definitions provided in prior systematic reviews [30, 31] and international guidelines, including those of the WHO and the ACSM [34, 35], to establish a unified classification framework. Based on these standards, exercise intensity was categorized into three levels: low, moderate, and moderate-to-high. Low-intensity exercise included light-load activities such as stretching, relaxation training, and certain mind–body practices. Moderate-intensity exercise encompassed brisk walking, aerobic routines, and moderate-load resistance training and was typically characterized by ranges such as 64%-76% maximum heart rate. Moderate-to-high intensity included higher-load resistance training, faster-paced running, and high-intensity interval training, corresponding to approximately 70%-85% maximum heart rate. For studies that reported heart rate or oxygen consumption ranges spanning two intensity categories (e.g., 40–65% heart rate reserve, 60–80% maximum heart rate), we applied a “dominant-range principle.” Under this rule, intensity was classified according to the category in which the majority of the reported range fell, while also considering the authors’ own descriptions of exercise intensity. For instance, although 40–65% heart rate reserve approaches the upper boundary of moderate-to-high intensity, most of the range corresponds to moderate intensity; therefore, it was coded as moderate. For trials that did not report objective intensity indicators such as heart rate, volume of oxygen consumption, or ratings of perceived exertion, intensity classification was determined based on exercise modality, workload characteristics, and prescription structure, following the same guideline-based criteria. This approach ensured consistency and comparability in intensity classification across studies with heterogeneous reporting practices.

In addition, because this study aimed to examine the dose-response relationship of exercise, total exercise duration and weekly exercise duration were calculated based on the exercise prescriptions reported in the original studies. Specifically, total exercise duration was computed as: Total duration = (exercise cycle) × (weekly frequency) × (session duration), and weekly exercise duration as: Weekly duration = (weekly frequency) × (session duration). Studies were included in the dose–response analysis only if they reported all three components necessary to calculate these dose metrics and provided outcome data that allowed the computation of change scores (post-pre). With respect to potential confounding variables, no additional covariate adjustments were made for factors such as exercise type, intensity, or sample characteristics. Instead, the three-level random-effects model was used to account for both within-study and between-study structural variability, allowing these differences to be incorporated as natural sources of heterogeneity. It should be noted, however, that the absence of explicit control for all potential confounders may affect the precision of the dose-response estimates. This limitation is acknowledged in the discussion section.

Two reviewers conducted All extraction and coding independently; disagreements were resolved by discussion with a third reviewer (XW) until consensus was reached.

### Risk of bias and certainty of evidence

We assessed risk of bias using the Cochrane Risk of Bias 2 (RoB 2) tool across five domains: randomization process; deviations from intended interventions; missing outcome data; measurement of the outcome; and selection of the reported result. Each domain was judged as low risk of bias, some concerns, or high risk of bias. An overall judgment of low risk of bias was assigned when all domains were rated low risk; some concerns when at least one domain was rated some concerns, and none was rated high risk; and high risk of bias when any single domain was rated high risk [39, 40].

Certainty of evidence was graded with GRADEpro. We downgraded certainty for risk of bias, inconsistency, indirectness, imprecision, and publication or reporting bias, and upgraded when appropriate [41]. Overall ratings were categorized as High, Moderate, Low, or Very Low. High indicates that further research is very unlikely to change confidence in the effect estimate; Moderate suggests that further research may have an important impact and may change the estimate; Low indicates that additional research is likely to have an important effect and is expected to change the estimate; and Very Low indicates that the effect estimate is very uncertain.

Any disagreements between the two reviewers (CL and RC) were resolved through discussion with a third reviewer (XW) until consensus was reached.

### Statistical analysis

Three-level meta-analyses were conducted in R using the metafor package, following the syntax described by Assink and Cui [42–45]. The primary purpose of the three-level random-effects model was to account for the dependency (non-independence) of multiple effect sizes extracted from the same study by partitioning variance across levels, rather than to control for confounding variables [46, 47]. Effect sizes were expressed as Hedges’ g (g) with 95% confidence intervals (CI) and 95% prediction intervals (PI) [48]. Overall heterogeneity was assessed with Cochran’s Q statistic. Variance components were estimated for level 1 sampling variance, level 2 within-study variance, and level 3 between-study variance, and their significance was tested with one-sided likelihood ratio tests (LRT) [49]. Sensitivity and influence analyses were used to evaluate the robustness of the findings and to identify high-influence estimates. Publication bias was examined using Egger’s test, funnel plots, and trim-and-fill procedures [50, 51].

Because moderators and dose were of interest, we performed subgroup and meta-regression analyses and modeled dose-response using total exercise dose and weekly exercise time as dose metrics [52]. We also conducted an exploratory mediation analysis within a three-level meta-analytic framework, estimating path a (exercise to cognition), path b(cognition to depressive symptoms), and path c’ (exercise to depressive symptoms). Further details are provided in Appendix 2.6.

The analytical workflow comprised: (1) pooling effect sizes for the impact of exercise on cognitive function; (2) heterogeneity testing; (3) subgroup analyses; (4) dose-response modeling for cognitive outcomes; (5) exploratory mediation analysis of the exercise–cognition–depressive symptoms relationship; (6) sensitivity and influence analyses; and (7) assessment of publication bias.

## Results

### Study selection

A total of 26,987 records were identified through six electronic databases (WOS, PubMed, the Cochrane Library, Embase, CNKI, and Wanfang), and an additional 21 records were identified from the reference lists of previous reviews, yielding 27,008 records in total. After removing 18,723 duplicates, 8,285 records were screened by title and abstract, of which 8,241 were excluded as irrelevant. We assessed 44 full-text articles for eligibility and excluded 13 that did not meet the inclusion criteria (details provided in Table 3). Ultimately, 31 studies were included in the meta-analysis (Fig. 1).

### Characteristics of included studies

A total of 31 studies published between 2001 and 2025 were included. By country, 10 were conducted in China [53–62], 6 in the United States [20, 21, 63–66], 5 in Germany [67–71], 3 in Denmark [72–74], 3 in India [75–77], and 1 each in New Zealand [78], Switzerland [79], Lithuania [80], and the Netherlands [79]. Across all studies, 2,434 participants with depression were enrolled, with women outnumbering men in most samples. Eight studies recruited inpatients, and 23 recruited outpatients. Twenty-eight were RCTs and three were nonRCTs. Baseline depression severity was most commonly assessed with the Beck Depression Inventory-II, Beck Depression Inventory, and the Hamilton Depression Rating Scale. Details are provided in Table 4.

In the intervention arms, 13 studies used exercise only, while 19 combined exercise with other therapies; one multi-arm trial included both an exercise-only group and an exercise-plus-other-therapy group [77]. Exercise type included aerobic (e.g., cycling, jogging, walking), resistance/strength training, mind–body practices (yoga or tai chi), and multicomponent exercises. Exercise frequency ranged from 1 to 5 sessions per week; intervention duration from 3 to 16 weeks; and exercise intensity was categorized as low, moderate, or moderate to high. Session duration ranged from 30 to 180 min. Control conditions included waitlist, treatment as usual, cognitive training, pharmacotherapy, and relaxation training. Additional intervention characteristics are summarized in Table 5.

### Risk of bias

**Fig. 2:**
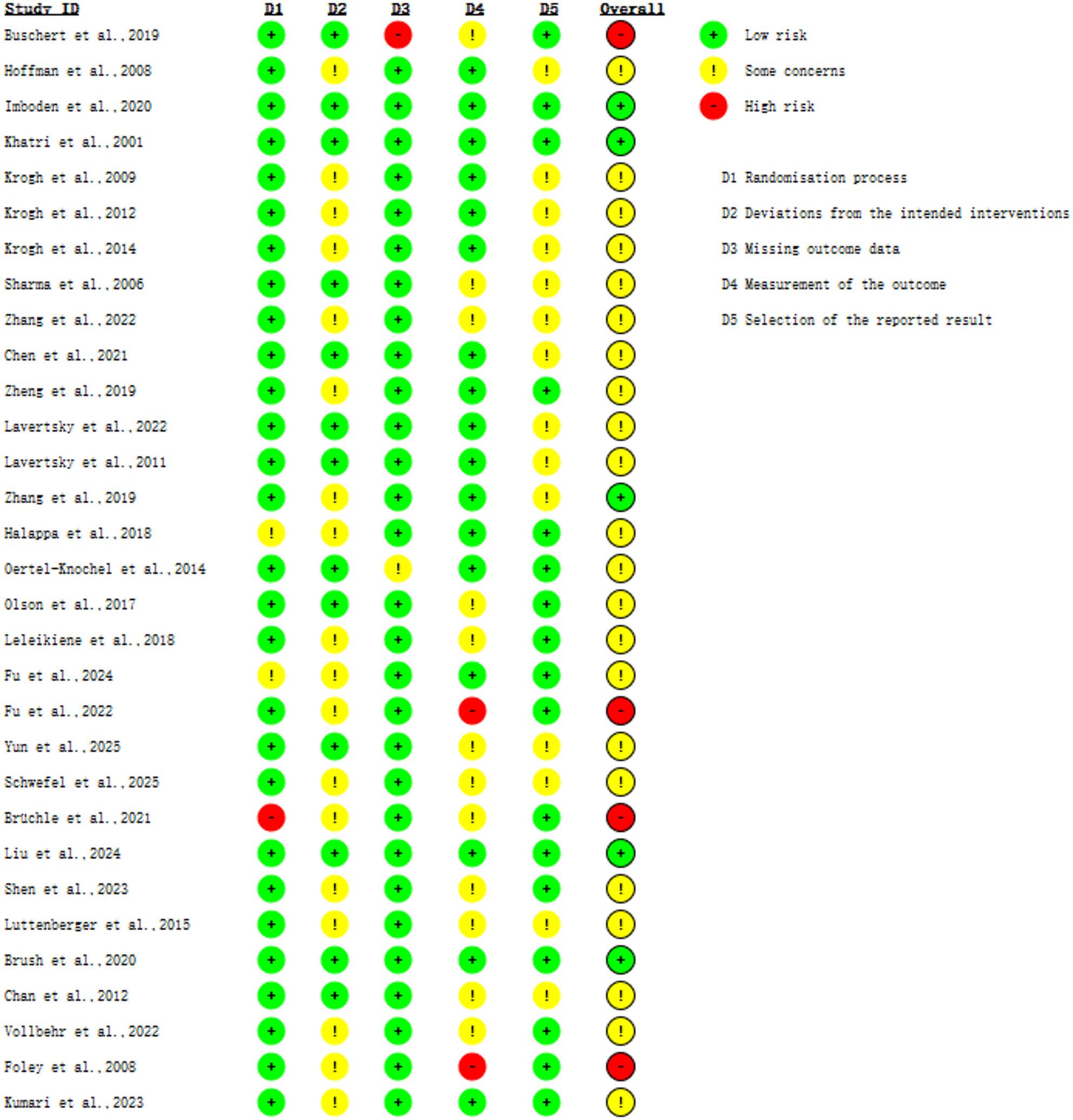
Risk of bias assessment of included studies

Across the 31 included studies, 28 were rated low risk for the randomization process, 13 for deviations from intended interventions, 29 for missing outcome data, 17 for measurement of the outcome, and 17 for selection of the reported result. Overall, 5 studies were judged to be at low risk of bias, 4 at high risk, and 22 with some concerns (Fig. 2).

### Meta-analytic results

We evaluated the effect of exercise on cognitive function in patients with depression. In the three-level random-effects model, the overall effect was significant (g = 0.24; 95% CI, 0.14 to 0.33; *p* < 0.001; 95% PI, -0.09 to 0.56; see Fig. 1 in the appendix). For comparison, a two-level random-effects model also yielded a significant overall effect (g = 0.20; 95% CI, 0.13 to 0.26; *p* < 0.001; 95% PI, 0.13 to 0.26).

### Heterogeneity analysis

Cochran’s Q statistic was not significant (Q[255] = 159.34, *p* > 0.49). The variance decomposition indicated that sampling variance (level 1) accounted for approximately 91.2%, within-study variance (level 2) for 1.7%, and between-study variance (level 3) for 8.8%. A LRT showed that the between-study variance (level 3) was statistically significant (LRT = 6.96, *p* = 0.004), indicating meaningful heterogeneity across studies and supporting the use of a three-level model rather than a two-level model. In contrast, the within-study variance (level 2) was not significant (LRT < 0.01, *p* > 0.49), suggesting minimal within-study heterogeneity. Despite the non-significant level-2 component, the three-level specification remains more appropriate because multiple effect sizes were extracted from the same studies; accordingly, all subsequent analyses used the three-level random-effects model.

### Moderator analyses

**Fig. 3:**
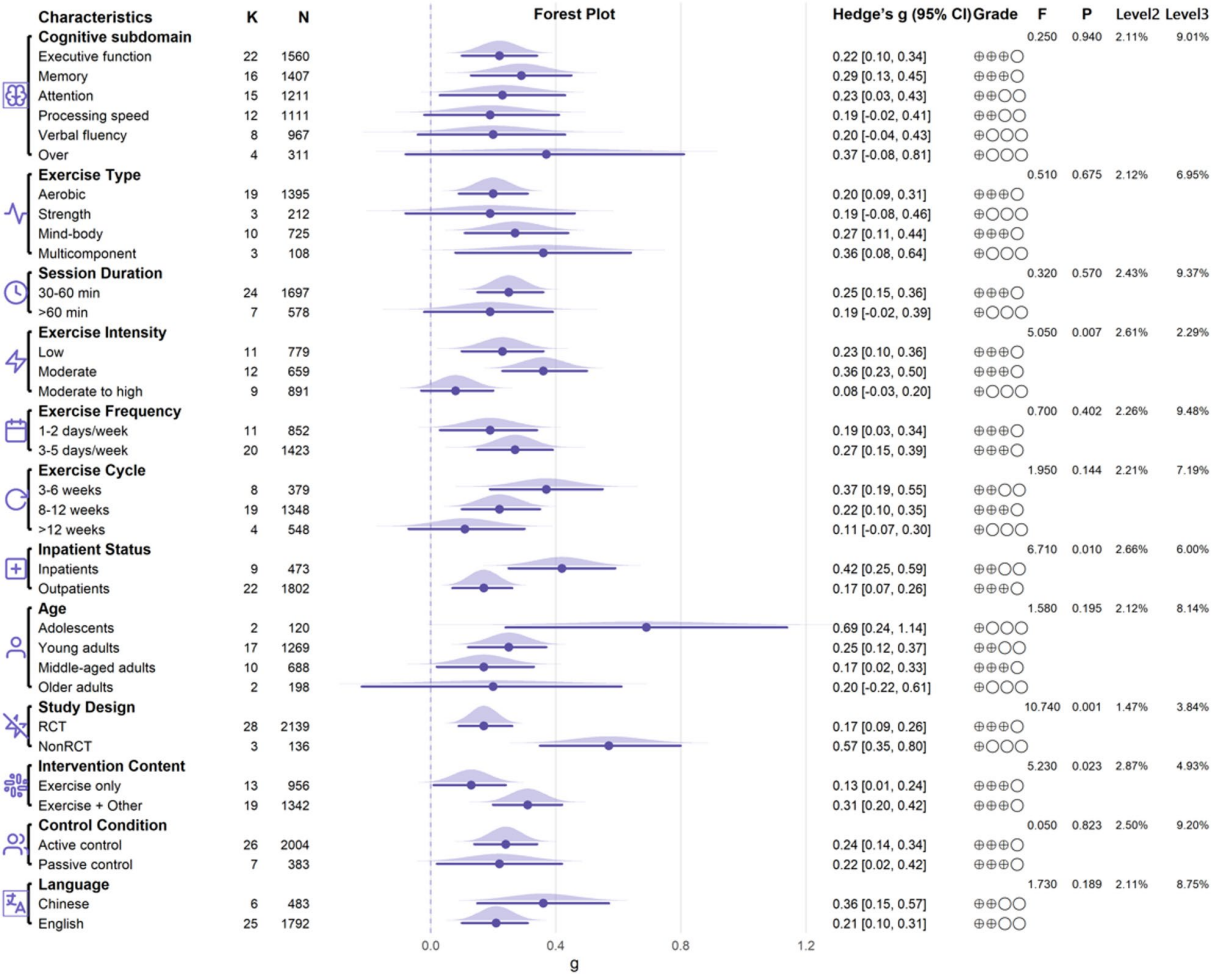
Subgroup analyses

Three-level meta-regressions identified several significant moderators. Exercise intensity (F(2,253) = 5.05, *p* = 0.007), inpatient status (F(1,254) = 6.71, *p* = 0.010), study design (F(1,252) = 10.79, *p* = 0.001), and intervention content (F(1,252) = 5.10, *p* = 0.025) significantly moderated the effect of exercise on cognition. Pairwise contrasts indicated that moderate intensity produced larger effects than moderate to high intensity (t = 3.15, *p* = 0.002), whereas low intensity did not differ from moderate to high intensity (t = 1.70, *p* = 0.090). Moderate versus low intensity was also not significantly different (t = 1.39, *p* = 0.165). In addition, effects were larger in inpatients than outpatients (t = 2.59, *p* = 0.010), larger in nonrandomized than randomized studies (t = 3.28, *p* = 0.001), and larger for exercise combined with other therapies than for exercise only (t = 2.29, *p* = 0.023). See Fig. 3; Table 5 in the appendix.

### Multiple moderator model

Using moderate to high intensity, exercise plus other therapies, nonRCT, and outpatient status as the reference categories, we fit a multivariable meta-regression. The overall moderator model was significant (F(5, 250) = 5.13, *p* < 0.001), indicating that at least one coefficient differed from zero. See Table 1.

**Table 1:** Multiple-moderator meta-regression results

Moderate	Group	k	β	95%CI	*p*
Exercise intensity	Intercept		0.36	0.05,0.68	0.025
	Low	11	0.10	-0.10,0.30	0.348
	Moderate	12	0.16	-0.04,0.37	0.120
Study type	RCT	28	-0.29	-0.50, -0.08	0.007
Intervention content	Exercise alone	13	-0.02	-0.22,0.18	0.837
Inpatient status	Inpatient	9	0.13	-0.09,0.34	0.245

k  number of studies, β  regression coefficient

### Dose–response relationships

For total exercise dose, the spline model showed no significant residual heterogeneity (QE(252) = 152.79, *p* > 0.49) and no overall effect (F(3,252) = 1.27, *p* = 0.286). A linear model likewise indicated no association between total dose and effect size (F(1,254) = 0.346, *p* = 0.557).

**Fig. 4:**
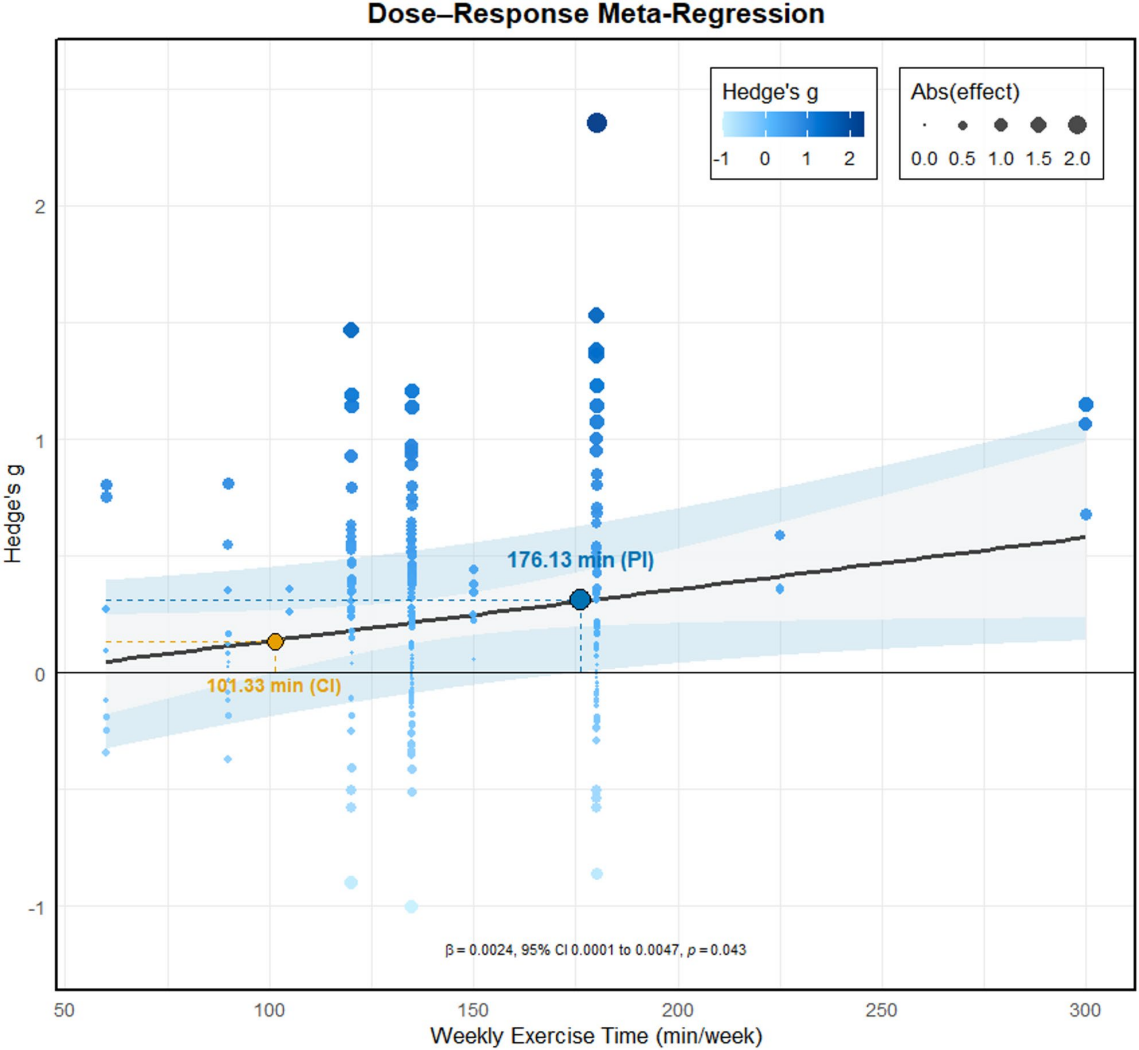
Relationship between weekly exercise time and the effect on cognitive function

For weekly exercise time, the spline model’s residual heterogeneity was not significant (QE(252) = 150.72, *p* > 0.49). The joint test of the spline terms was not significant (F(3,252) = 2.04, *p* = 0.109), although the third spline coefficient reached significance (β = 0.81, SE = 0.33, *p* = 0.016), providing no robust evidence for a nonlinear pattern. In contrast, a linear model indicated a positive association between weekly minutes and effect size (F(1,254) = 4.13, *p* = 0.043). The lower bound of the 95% CI crossed above zero at 101.33 min per week, suggesting that statistically detectable benefits begin around this level. When between-study heterogeneity was incorporated to derive 95% PIs, the lower bound exceeded zero at 176.13 min per week, indicating that future studies would be expected to observe positive effects at or above this weekly duration. See Fig. 4.

### Mediation analysis

Within a three-level meta-analytic framework, we specified a mediation model with cognitive function as the mediator and estimated the path a, path b, path c’. The indirect effect (a × b) and the proportion mediated were derived using the product-of-coefficients (Delta) method, with small-sample robustness checks via cluster-robust variance estimation (CR1), Knapp–Hartung tests for REML fits, and maximum-likelihood z-tests.

Path a: β = 0.13, SE = 0.07, 95% CI − 0.01 to 0.26, p = 0.068; CR1 p = 0.018; KH p = 0.095; ML(z) p = 0.068 (mixed significance across corrections). Path b : β = 0.15, SE = 0.04, 95% CI 0.07 to 0.23, p < 0.001; CR1 p = 0.004; KH p = 0.003; ML(z) p < 0.001 (significant). Path c’: β = 0.36, SE = 0.17, 95% CI 0.07 to 0.69, *p* = 0.034; CR1 *p* = 0.076; KH *p* = 0.078; ML(z) *p* = 0.019 (mixed significance across corrections). Indirect effect: a × b = 0.02, 95% CI − 0.00 to 0.04 (not significant); proportion mediated = 0.053 (5.3%). Taken together, no significant indirect effect via cognition was observed. Current evidence does not support cognition as a primary mediating pathway through which exercise influences depressive symptoms, and this finding should be interpreted with caution.

### Sensitivity and influence analyses

We used influence diagnostics to evaluate whether outliers affected the results and conducted leave-one-study-out (LOSO) and leave-one-effect-out (LOO) analyses to test dependence on any single study or effect. In the primary model for the effect of exercise on cognition, the largest deleted standardized residual reached |rstudent| = 3.41, whereas DFFITS ≤ 0.17, Cook’s D ≤ 0.03, leverage ≈ 0.002 to 0.007, and the covariance ratio ≈ 1 [71], indicating negligible influence of any single effect size (see Supplementary Fig. 2). Using the three-level random-effects model with REML as the baseline, the pooled effect was β = 0.24 (95% CI 0.15 to 0.33). Results were unchanged with CR2 robust standard errors and Satterthwaite degrees of freedom: β = 0.24 (95% CI 0.14 to 0.33), both significantly positive. In LOSO (CR2), removing any one study yielded β values ranging from 0.20 to 0.25; the maximum absolute change was |Δ| = 0.04 [71], about 18% of the baseline estimate (0.04/0.24), with the direction consistently positive. In LOO (CR2), the maximum absolute change was only |Δ| = 0.01 [71], about 4.6% of the baseline, indicating negligible impact. Overall, the primary model was not sensitive to the removal of individual studies or effects.

For the mediation components, influence diagnostics showed the following patterns. Path a: No observations exceeded thresholds (|rstudent| ≤ 2.20; DFFITS and Cook’s D below their cutoffs). In LOSO, pooled β ranged from 0.09 to 0.15; occasional CIs crossed zero, but the direction was unchanged. Path b: DFFITS identified three high-influence candidates [78, 79]; for one effect size [78], Cook’s D slightly exceeded the conventional cutoff, but none met outlier criteria (|rstudent| < 3). LOSO produced pooled β values from 0.13 to 0.17, leaving conclusions unchanged. Path c’: One study [55] exceeded thresholds on rstudent, DFFITS, and Cook’s D. Excluding this study reduced β from 0.36 to 0.16 (95% CI 0.01 to 0.32) without changing the effect’s direction; LOSO yielded a range of 0.16 to 0.43, indicating that isolated high-influence observations can attenuate the magnitude but do not reverse the direction of the effect.

### Publication bias

**Fig. 5:**
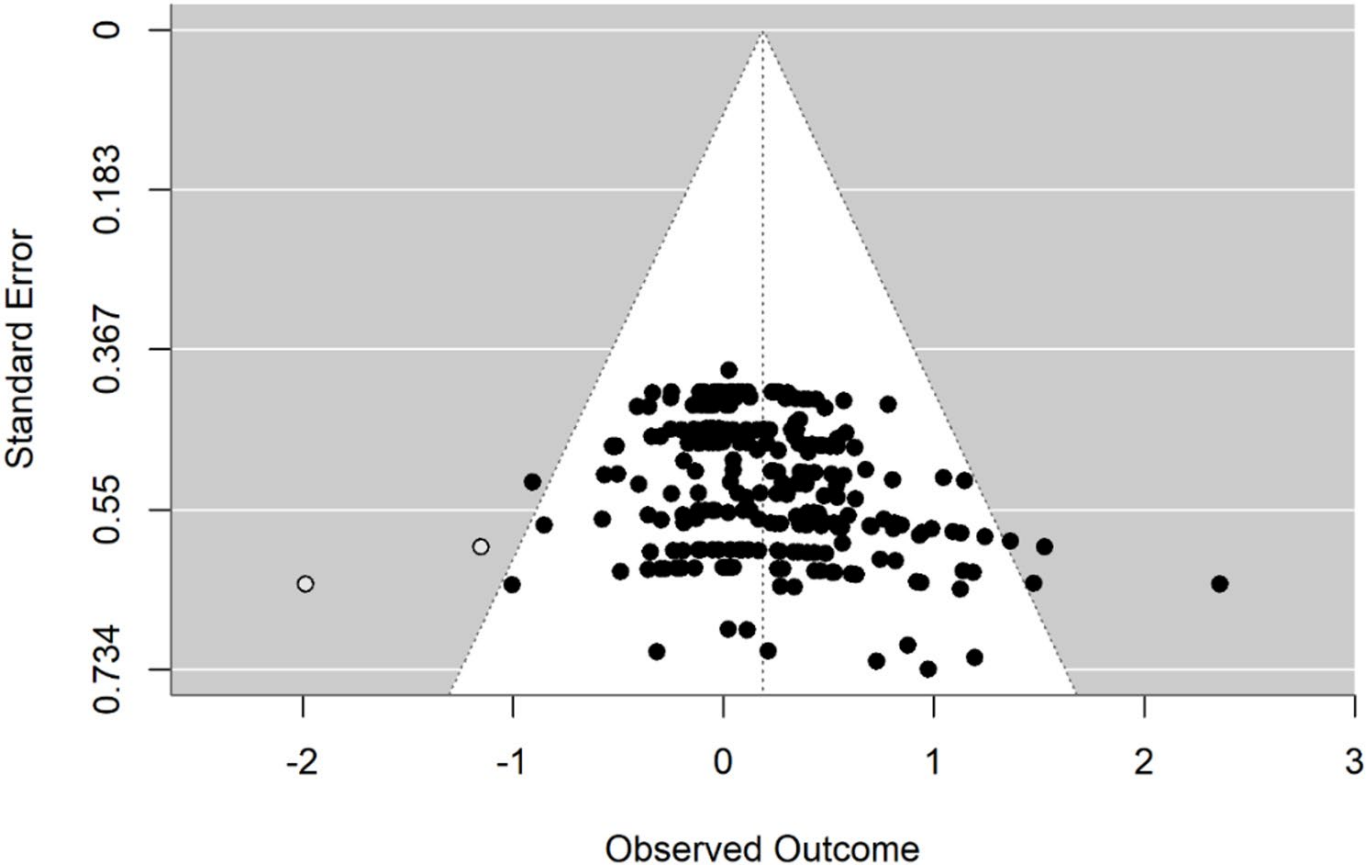
Funnel plot for the effect of exercise on cognitive function in patients with depression (after trim-and-fill)

We examined publication bias using funnel plots, two-level and three-level Egger regressions, and the trim-and-fill procedure to assess small-study effects. The funnel plot appeared asymmetric (Fig. 5). Egger tests indicated small-study bias in both the two-level model (t(254) = 4.75, *p* < 0.001) and the three-level model (slope = 1.66, SE = 0.67; t(11.0) = 2.46, *p* = 0.032; CR2 small-sample correction). However, when predictors (low or moderate intensity, exercise-only, inpatient status, and RCT) were added to the three-level Egger regression, the association was no longer significant (slope = 0.35, SE = 0.80; t(11.2) = 0.44, *p* = 0.668), suggesting that the observed funnel asymmetry may be partly explained by these study characteristics. Trim-and-fill estimated two missing effects on the left side of the funnel; after imputation, the adjusted overall effect remained significant (g = 0.19; 95% CI 0.12 to 0.25) and was close to the observed estimate (g = 0.20; 95% CI 0.13 to 0.26). Overall, despite evidence of small-study bias, the results were robust to bias adjustment.

For the mediation components, publication bias signals were pathway-specific. Path a: No evidence of bias (two-level Egger p = 0.969; trim-and-fill added 0 studies on either side; three-level Egger with CR2 slope p = 0.958). Path b: Evidence of bias (two-level Egger p < 0.001). Trim-and-fill suggested 7 missing studies on the left; the pooled effect decreased from g = 0.13 (95% CI 0.04 to 0.21) to g = 0.03 (95% CI − 0.08 to 0.13). The three-level Egger (CR2) also indicated a significant slope on the standard error (p < 0.001). Path c’: Little or inconsistent evidence of bias (two-level Egger *p* = 0.055; trim-and-fill added 1 study on the right, yielding g = 0.42, 95% CI 0.11 to 0.73; three-level Egger CR2 slope *p* = 0.253).These pathway-specific findings should be interpreted cautiously. Further details are provided in Supplementary Figs. 3–4.

### Certainty of evidence

Using GRADE, we rated the certainty of evidence for the primary model, all subgroup analyses, and the three mediation paths. Overall, certainty was moderate for the primary effect of exercise on cognition and for the following subgroups: executive function, memory, aerobic exercise, mind-body exercise, session duration 30 to 60 minutes, low intensity, moderate intensity, 1 to 2 days per week, 3 to 5 days per week, outpatients, middle aged adults, RCTs, exercise cycle 8 to 12 weeks, exercise only, exercise plus other therapies, active control, and passive control. Certainty was low for attention, processing speed, exercise cycle 3 to 6 weeks, inpatients, young adults, Chinese language studies, English language studies, path a, and path c’. Certainty was very low for verbal fluency, global cognition, strength training, multicomponent exercise, session duration greater than 60 min, moderate to high intensity, exercise cycle greater than 12 weeks, adolescents, older adults, nonRCTs, and path b. Detailed ratings are provided in Table 6.

## Discussion

We included 31 studies to evaluate the effects of exercise on cognitive function in depression, examine moderators, and characterize an optimal dose; in addition, five studies explored the exercise–cognition–depression linkage using mediation approaches. Overall, exercise was associated with improvements in cognitive function. Moderator analyses indicated that exercise intensity, inpatient status, study design, and intervention content systematically influenced effect sizes. However, the available evidence did not support cognition as a primary mediating pathway through which exercise reduces depressive symptoms, and this finding should be interpreted cautiously. Dose-response analyses identified a minimum effective weekly duration, with cognitive benefits becoming more likely once weekly exercise exceeded approximately 176 min; however, this represents a model-based threshold rather than a universal optimal dose.

### Effects of exercise on global and domain-specific cognition in depression

Exercise produced a statistically significant small-to-moderate improvement in cognitive function among individuals with depression. This aligns with the three level meta analysis by Ren et al., which synthesized 22 RCTs and reported similar benefits. Unlike that review, the present study also included quasi experimental trials and Chinese language publications, thereby increasing the evidence base and statistical power. In addition, we extracted change scores (post minus pre) rather than relying solely on post intervention means, a strategy that can mitigate baseline imbalance and often yields greater precision [81]. Methodologically, our use of a three level model allowed inclusion of multiple correlated outcomes per study while accounting for dependence, reducing unit of analysis bias and improving inferential reliability [42]. Not all prior syntheses agree. Sun et al. pooled 12 randomized and 3 quasi experimental trials and found no improvement in global cognition or cognitive subdomains [82]; similarly, Brondino et al. reported null effects across eight RCTs [33]. Relative to these reviews, our meta analysis incorporated more studies and effect sizes and modeled within study clustering directly, which may explain the discrepant conclusions. Potential mechanisms are consistent with neurobiological models of exercise induced cognitive change. In patients with depression, a 12 week exercise program has been associated with increased left hippocampal activation accompanied by improvements in working memory [68]. Exercise may also upregulate hippocampal brain derived neurotrophic factor, and promote cortical capillary angiogenesis, processes linked to cognitive enhancement [83, 84].

At the domain level, exercise was associated with improvements in executive function, attention, and memory, but not in verbal fluency or processing speed. One interpretation is that early cognitive gains emerge in networks subserving executive control and memory-particularly fronto-hippocampal pathways-whereas other domains may require more targeted cognitive training to change [68, 85]. Our findings are not fully concordant with Ren et al., who also reported benefits in processing speed [31] likely source of discrepancy is heterogeneity in domain definitions and task mapping, differences in instrument sensitivity and scoring approaches, and variation in the composition of the evidence base [86]. Future trials and reviews should standardize domain mapping and scoring and compare domain specific responsiveness under matched exercise dose and measurement conditions.

### Moderators

#### Characteristics of exercise interventions

In our analyses, exercise type, session duration, exercise frequency, and exercise cycle were not significant moderators, whereas exercise intensity emerged as an important moderator of cognitive outcomes in depression. This pattern is not fully consistent with prior syntheses: Ren et al. identified session duration within aerobic programs as a moderator [31, 86], while Liu et al. reported that intensity moderated exercise effects on working memory in depression [16].

Regarding intensity, both low and moderate intensities were associated with significant benefits, and moderate intensity differed significantly from moderate to high intensity. These findings diverge from two recent reviews [31, 86]. Discrepancies may reflect differences in the number of included studies, coding schemes, and the mix of intervention types across evidence bases. Mechanistically, the apparent advantage of moderate intensity may relate to more efficient catecholamine release, greater central arousal, and improved allocation of cognitive resources [87]. That said, some work favors higher intensities: Greer et al. reported superior improvements in visuospatial learning and memory with high-intensity aerobic exercise versus low intensity among patients with depression [88], and other reviews have suggested benefits at the lower end of the intensity spectrum [82]. Taken together, intensity likely exerts a nontrivial influence, but the optimal range may depend on population and task demands.

For type, aerobic, mind–body, and multicomponent exercise were associated with cognitive improvement, whereas strength training showed no clear benefit. This may be a function of limited evidence: only three strength-training studies met inclusion, and one reported low adherence (50%) [72], a factor known to attenuate observable cognitive gains [82]. For frequency, both 1 to 2 days per week and 3 to 5 days per week were associated with benefits. Differences from earlier reviews likely stem from alternative frequency bins (e.g., 2 vs. 3 sessions per week [86], or 1, 2, and ≥ 3 sessions per week [31]. For session duration, we observed benefits for 30 to 60 min per session, whereas more than 60 min did not show additional advantage. Ren et al. previously reported improvements at both durations within aerobic training [86], although their more recent analysis also noted limited effects when sessions exceeded 60 min [31]. Prolonged sessions may increase fatigue or dehydration [89], and from a self-control perspective, long or very intense bouts may deplete regulatory resources, potentially impairing subsequent cognitive task performance [90]. For exercise cycle, programs of 3 to 6 weeks and 8 to 12 weeks were effective, whereas those longer than 12 weeks were not. Ren’s most recent work used a 10-week cut point and reported a different pattern [31]; our categorization differs, and the > 12-week subgroup in our dataset contained only four studies, several with suboptimal adherence. More long-duration trials with careful adherence monitoring are needed to clarify duration effects.

#### Sample and study characteristics

Inpatient status, study design, and intervention content emerged as significant moderators, whereas age, control condition, and publication language did not moderate the effect.

For inpatient status, exercise improved cognition in both inpatients and outpatients, with larger effects in inpatients, consistent with prior work [31]. Greater supervision, higher adherence, and a more consistent delivered dose in inpatient settings likely enhance outcomes, whereas higher attrition and implementation variability in outpatient contexts may dilute true effects [91, 92]. By study design, both RCTs and nonRCTs yielded significant benefits, but effects were larger in nonRCTs. This pattern is compatible with the possibility that residual confounding and performance or detection biases in nonrandomized designs may inflate effect sizes relative to more rigorous RCTs [93]. Regarding intervention content, exercise only and exercise plus other therapy both improved cognition, with combined interventions outperforming exercise alone. Add-on components such as cognitive training or psychotherapy may target cognitive pathways more directly, producing greater gains [94]. Notably, some reviews have reported that exercise alone may be insufficient to improve cognition in depression [82].

Across age strata, benefits were evident for adolescents, young adults, and middle-aged adults, but not for older adults, broadly aligning with previous findings [31]. In analyses by control condition, exercise showed advantages versus both active and passive controls. Effects were also observed in studies published in English and Chinese, indicating that language of publication did not account for between-study differences.

### Dose-response between exercise and cognition in depression

Total exercise dose was not a significant moderator, consistent with prior work [31]. Notably, we identified weekly exercise time as an important moderator.Based on the dose-response curve, cognitive benefits were unlikely when weekly duration was below approximately 101.33 min, whereas improvements began to appear more consistently once weekly duration exceeded roughly 176.13 min. Importantly, this threshold does not represent a universal optimal dose. Rather, it reflects a model-based trend derived from aggregated data across heterogeneous exercise types. Different modalities-such as aerobic, resistance, and mind-body exercise-differ substantially in physiological load, efficiency, and time structure, and may therefore reach effective doses at different weekly durations. Resistance training and high-intensity exercises, for instance, often involve shorter durations yet still produce meaningful cognitive gains. Thus, the identified threshold should be interpreted as an indicative minimum weekly volume at which cognitive improvements become more likely in mixed-modality analyses, not as a prescriptive recommendation or maximal dose. Future work should examine whether dose thresholds vary across exercise types and intensities. This aligns with broader guidance: the WHO recommends 150 to 300 min of moderate intensity activity per week for health gains [18], and cognitive epidemiology indicates a lower threshold around 700 metabolic equivalent of task minutes per week (approximately 150 to 180 min of moderate intensity) [95]. Mechanistically, shorter weekly exposure may be insufficient to induce structural and functional brain adaptations linked to cognitive improvement [96, 97].

### Mediation analysis

To our knowledge, this is the first exploratory synthesis of the exercise–cognition–depressive symptoms pathway within a three-level meta-analytic framework. We observed a significant b path and evidence for a direct c′ path, whereas the indirect effect via cognition was small and not significant (a × b 95% CI crossed zero; proportion mediated ≈ 5%). This pattern suggests that cognitive function may not represent the primary mechanism through which exercise alleviates depressive symptoms. Several factors may account for this finding. First, cognitive enhancement typically requires relatively long intervention periods and sufficiently high training doses. However, most included studies implemented programs lasting only 4–12 weeks, which may be inadequate for producing the degree of cognitive change necessary to yield a detectable mediation effect. Second, substantial heterogeneity existed in cognitive assessment tools across studies. Variations in sensitivity, scoring methods, and targeted domains (e.g., executive function, processing speed, attention) may have reduced the statistical power to detect mediation. Third, the mechanisms through which exercise improves depressive symptoms are inherently multifaceted; cognitive improvement may constitute only a relatively weak pathway. Similar patterns have been reported previously: in an RCT, 8 weeks of aerobic exercise improved depressive symptoms and modulated neural markers of cognitive control (e.g., N200 amplitude), yet changes in N200 did not mediate the reduction in depressive severity [63]. Other studies have even suggested that depressive symptoms may serve as a mediator in the relationship between physical activity and cognition, rather than the reverse [98].

Beyond cognition, exercise may influence depressive symptoms more directly through alternative pathways. Exercise has been shown to improve emotion regulation capacity, reduce inflammatory processes, enhance neuroplasticity (e.g., increased brain-derived neurotrophic factor), improve sleep quality, and promote self-efficacy and social interaction-all plausible and potentially stronger mediators than cognitive function. Thus, although the present findings do not support cognition as the primary mechanism, this does not eliminate the possibility that cognition plays an auxiliary role. Future research should employ more sensitive cognitive measures, longer intervention durations, and study designs that simultaneously incorporate key mechanistic variables such as emotion regulation, biological markers, sleep parameters, and self-efficacy. Such work will be essential for constructing a more comprehensive mechanistic framework describing how exercise reduces depressive symptoms.

### Strengths and limitations

This review has several strengths. (1) To our knowledge, it is the first synthesis to apply a three level meta analytic approach that integrates both Chinese and English literature and includes randomized and nonrandomized studies, spanning inpatient and outpatient settings, active and passive controls, and exercise only versus exercise plus other therapy-thereby enlarging the evidence base and improving generalizability. (2) It is the first to examine weekly exercise time as a dose metric for cognitive outcomes in depression, providing a pragmatic target for future protocols. (3) It is also the first to explore the exercise–cognition–depressive symptoms pathway within a three level framework.

Naturally, this study has several limitations. (1) The included populations with depression varied in diagnostic criteria and assessment tools; therefore, we did not conduct subgroup analyses based on depressive-symptom severity, which may obscure true differences across subgroups. (2) Cognitive function was assessed using diverse instruments and task paradigms. These tools differed in sensitivity, scoring methods, and the specific cognitive domains they covered, which may introduce measurement error and affect the stability of the estimated effects. (3) Regarding the dose-response analyses, weekly and total exercise durations were calculated based on the prescribed exercise programs reported in the original studies; however, the actual exercise dose completed by participants may not have fully matched the prescribed dose. Moreover, the dose–response models did not explicitly control for potential confounding factors such as exercise type, intensity, or sample characteristics. As such, the precision of the dose–response estimates should be interpreted with caution. (4) Although we included both RCTs and non-RCTs to broaden the evidence base, variability in methodological quality-particularly in randomization procedures, allocation concealment, and blinding-may have influenced effect-size estimates. (5) The exploratory mediation analysis investigating the exercise–cognition–depression pathway was limited by the small number of eligible studies, as well as heterogeneity in assessment tools and measurement time points; therefore, these mediation findings should also be interpreted cautiously. (6) Although we broadened our search to include Chinese-language databases to reduce the risk of language bias, the inclusion of Chinese studies alone cannot fully eliminate this bias. On the one hand, Chinese databases expand coverage by capturing relevant studies from non-English contexts; on the other hand, reporting standards in some Chinese journals differ from those of international journals, and methodological details (e.g., randomization procedures, blinding, sample size estimation) may be insufficiently reported, potentially affecting the accuracy of risk-of-bias assessments. We therefore applied strict predefined inclusion criteria and used the RoB 2 tool to rigorously evaluate methodological quality. Nevertheless, we acknowledge that differences in reporting standards across languages may still influence overall effect estimates, representing another limitation of this study.

### Future directions

Future work should harmonize diagnostic criteria and adopt standardized cognitive assessment batteries to reduce measurement related heterogeneity. Trials should document actual exercise dose and adherence throughout implementation to enable more precise dose response modeling. Study conduct should emphasize allocation concealment, blinding, and rigorous handling of missing data, and whenever feasible provide secondary analyses with sufficient detail on covariates and correlation or covariance structures to permit more definitive tests of mediation mechanisms.

## Conclusion

Exercise improves cognitive function in patients with depression. Rather than indicating a universal optimal dose, our dose-response findings suggest a minimum weekly activity threshold, with cognitive benefits becoming more likely when moderate-intensity exercise exceeds approximately 176 min per week. Present evidence does not support cognition as the primary mediator of the exercise-depression relationship, and this finding should be interpreted cautiously. Future multicenter RCTs with standardized outcome measures and modality-specific dose-response analyses are needed to refine the minimum effective dose and clarify potential mediating pathways.

## Supplementary Information

Supplementary Material 1.

Supplementary Material 2.

## Data Availability

All data generated or analyzed in this three-level meta-analysis are provided in the article and its Supplementary Information and have been deposited in the Open Science Framework (OSF) repository.

## Abbreviations

ACSM: American College of Sports Medicine
CI: Confidence Interval
CR2: Cluster-Robust Variance Estimator, Type 2
Cook’s D: Cook’s Distance
CNKI: China National Knowledge Infrastructure
DFFITS: Difference in Fits
DSM: Diagnostic and Statistical Manual of Mental Disorders
g: Hedges’ g
GRADE: Grading of Recommendations, Assessment, Development, and Evaluations
ICD: International Classification of Diseases
LRT: Likelihood ratio tests
LOSO: Leave-one-study-out
LOO: Leave-one-effect-out
PI: Prediction intervals
PRISMA: Preferred Reporting Items for Systematic Reviews and Meta Analyses Statement
PROSPERO: International Prospective Register of Systematic Reviews Analyses Statement
RCTs: Randomized controlled trial
RoB 2: Cochrane Risk of Bias tool
USD: United States dollar
WHO: World Health Organization
WOS: Web of Science
