# Supplementary material for: Effects of exercise on cognitive function in patients with depression: a three-level meta-analysis with dose-response and exploratory mediation analyses
Source: Int J Behav Nutr Phys Act. 2026 Mar 11;23:38. doi: 10.1186/s12966-026-01902-3 (PMC13097831; doi:10.1186/s12966-026-01902-3)
Supplement: Supplementary file 1 — Supplementary Material 1. [file 12966_2026_1902_MOESM1_ESM.docx]

**Supplementary material** for

**Effect of exercise on cognitive function in patients with depression: a three-level meta-analysis with dose-response and exploratory mediation analyses**

**2.2 Literature search strategy**

Table 1.Literature Search Strategy

| Databases | Search Strategy | Filters/limitations |
| --- | --- | --- |
| Pubmed  (N=4822) | #1 "Exercise"[Mesh]  #2 "Aerobic exercise" [Title/Abstract] OR "Resistance exercise"[Title/Abstract] OR "High-intensity interval" [Title/Abstract] OR "Yoga" [Title/Abstract] OR "Dance" [Title/Abstract] OR "Taichi" [Title/Abstract] OR "Baduanjin" [Title/Abstract] OR "Wuqinxi" [Title/Abstract] OR "Yijinjing" [Title/Abstract] OR "Walking" [Title/Abstract] OR "mind-body exercise" [Title/Abstract] OR "exercise"[Title/Abstract] OR “physical activity”[Title/Abstract]  #3 "Depressive Disorder"[Mesh] OR "Depressive Disorder, Major"[Mesh]  #4 "Depressive disorder" [Title/Abstract] OR "Depressive symptom" [Title/Abstract] OR "Emotional depression" [Title/Abstract] OR "Depressive neurosis" [Title/Abstract] OR"Endogenous depression" [Title/Abstract] OR "Deurotic depression" [Title/Abstract] OR "Unipolar depression" [Title/Abstract] OR "Major Depressive Disorder"[Title/Abstract] OR "Depression"[Title/Abstract]  #5 "Cognition"[Mesh]  #6 "Cognition"[Title/Abstract] OR “Executive function”[Title/Abstract] OR “Cognitive performance” [Title/Abstract] OR “Cognitive” [Title/Abstract] OR “Working memory” [Title/Abstract] OR “Shifiting” [Title/Abstract] OR “Inhibitory function”[Title/Abstract]  #7 #1 OR #2  #8 #3 OR #4  #9 #5 OR #6  #10 #7 AND #8 AND #9 | English |
| Embase  (N=9472) | ('depression'/exp OR 'major depression'/exp OR 'depressive disorder':ti,ab OR 'depressive symptom':ti,ab OR 'emotional depression':ti,ab OR 'depressive neurosis':ti,ab OR 'endogenous depression':ti,ab OR 'deurotic depression':ti,ab OR 'unipolar depression':ti,ab OR 'major depressive disorder':ti,ab OR 'depression':ti,ab) AND ('exercise'/exp OR 'aerobic exercise':ti,ab OR 'resistance exercise':ti,ab OR 'high-intensity interval':ti,ab OR 'yoga':ti,ab OR 'dance':ti,ab OR 'taichi':ti,ab OR 'baduanjin':ti,ab OR 'wuqinxi':ti,ab OR 'yijinjing':ti,ab OR 'walking':ti,ab OR 'mind-body exercise':ti,ab OR 'exercise':ti,ab OR 'physical activity':ti,ab) AND ('cognition'/exp OR 'cognition':ti,ab OR 'executive function':ti,ab OR 'cognitive performance':ti,ab OR 'cognitive':ti,ab OR 'working memory':ti,ab OR 'shifiting':ti,ab OR 'inhibitory function':ti,ab | Article, Article in Press, Preprint/English |
| Web of Science (N=5154) | #1 TS=(“Aerobic exercise” OR “Resistance exercise” OR “High-intensity interval” OR “Yoga” OR “Dance” OR “Taichi” OR “Baduanjin” OR “Wuqinxi” OR “Qigong” OR “Walking” OR "Yijinjing" or "mind-body exercise" OR "exercise" OR “physical activity”)  #2 TS=("Depressive disorder" OR "Depressive symptom" OR "Emotional depression" OR "Depressive neurosis" OR "Endogenous depression" OR "Deurotic depression" OR "Unipolar depression" OR "Major Depressive Disorder" OR "Depression")  #3 TS=("Cognition" OR “Executive function” OR “Cognitive performance” OR “Cognitive” OR “Working memory” OR “Shifiting” OR “Inhibitory function”)  #4 #1 OR #2 OR #3 | Article, Proceeding paper/ English |
| the Cochrane Library (N=4754) | #1 "Exercise"[Mesh]  #2 "Aerobic exercise" [Title/Abstract] OR "Resistance exercise"[Title/Abstract] OR "High-intensity interval" [Title/Abstract] OR "Yoga" [Title/Abstract] OR "Dance" [Title/Abstract] OR "Taichi" [Title/Abstract] OR "Baduanjin" [Title/Abstract] OR "Wuqinxi" [Title/Abstract] OR "Yijinjing" [Title/Abstract] OR "Walking" [Title/Abstract] OR "mind-body exercise" [Title/Abstract] OR "exercise"[Title/Abstract] OR “physical activity”[Title/Abstract]  #3 "Depressive Disorder"[Mesh] OR "Depressive Disorder, Major"[Mesh]  #4 "Depressive disorder" [Title/Abstract] OR "Depressive symptom" [Title/Abstract] OR "Emotional depression" [Title/Abstract] OR "Depressive neurosis" [Title/Abstract] OR"Endogenous depression" [Title/Abstract] OR "Deurotic depression" [Title/Abstract] OR "Unipolar depression" [Title/Abstract] OR "Major Depressive Disorder"[Title/Abstract] OR "Depression"[Title/Abstract]  #5 "Cognition"[Mesh]  #6 "Cognition"[Title/Abstract] OR “Executive function”[Title/Abstract] OR “Cognitive performance” [Title/Abstract] OR “Cognitive” [Title/Abstract] OR “Working memory” [Title/Abstract] OR “Shifiting” [Title/Abstract] OR “Inhibitory function”[Title/Abstract]  #7 #1 OR #2  #8 #3 OR #4  #9 #5 OR #6  #10 #7 AND #8 AND #9 | English |
| CNKI (N=842) | #1 运动 + 体育锻炼 + 太极 + 瑜伽 + 气功  #2 抑郁症 + 抑郁  #3 认知功能 + 认知 + 工作记忆 + 执行功能 + 抑制  #4 #1 AND #2 AND #3 |  |
| Wanfang (N=1943) | #1 运动 OR 体育锻炼 OR 太极 OR 瑜伽 OR 气功  #2 抑郁症 OR 抑郁  #3 认知功能 OR 认知 OR 执行功能 OR 工作记忆 OR抑制  #4 #1 AND #2 AND #3 |  |

CNKI for China National Knowledge Infrastructure

**2.4 Data extraction and coding**

Table 2. Selected Coding Information

| Moderators | Code | Specific modality |
| --- | --- | --- |
| Control Group | Active control | Light stretching,mental relaxation training, The cognitive game, occupational or art therapy,stretching,relaxation training, sertraline,Light stretching,Escitalopram combined with health education, Antidepressants,conventional antidepressant treatment, conventional rehabilitation |
|  | Passive control | Waitlist,sit quietly，placebo pill |
| Cognitive subdomain | Executive function | Flankers task，letter–number–span,Spatial Span, Trail making test-B, Go-Nogo, Tower of London, N-back, Stroop, Digit span test, standard progressive matrices, Computergestütztes Kartensortierverfahren,working memory difficulty level 3， flexibility-verbal change, N-back, Ruff Figural Fluency Test, ,the Wisconsin Card Sorting Test |
|  | Memory | Hopkins Verbal Learning Test-Revised,Brief Visuospatial Memory Test-Revised, verbal learning and memory test,un-cued recall, Rey auditory verbal learning test，Verbal Paired Associates Subtest from the Wechsler Memory Scale，Logical Memory Subtest from the Wechsler Memory Scale,Visual Reproduction Associates Subtest from the Wechsler Memory Scale, the Buschke Test, the Rey Complex Figure Test, the California Verbal Learning Test II |
|  | Attention | Work performance series, Testbatteriezur Aufmerksamkeitsprüfung，Digit Vigilance  Test,Subtracting Serial Sevens, the matching grids test, the mathematical processing test, the 2-choice reaction time test, the simple reaction time test, Letter Cancellation Test, Continuous performance task,Exogenous Cueing Task,d2-R |
|  | Verbal fluency | Animal Naming，Controlled Oral Word Association Test,, Animal Fluency, Boston Naming Test |
|  | Processing speed | Trail Making Test-A, Symbol Coding,Digit Symbol Test，Ruff 2 & 7 Test |
|  | Over | Mini-Mental State Examination,the Montreal Cognitive Assessment,Repeatable Battery for the  Assessment of Neuropsychological Status |

**2.6 Statistical analysis**

We extracted change scores (means and standard deviations) for cognitive outcomes in the intervention and control groups from the included studies and converted them to effect sizes and corresponding variances [1, 2]. All effect-size synthesis, sensitivity and influence analyses, subgroup analyses, publication-bias assessments, and mediation (structural-equation) analyses were performed in R, following the syntax and workflows described by Assink and Cui [3, 4].

Because many studies reported multiple cognitive outcomes (e.g., Stroop accuracy and reaction time), we used a three-level meta-analytic model that partitions variance into sampling variance (level 1), within-study variance (level 2), and between-study variance (level 3). This approach enables aggregation of all relevant cognitive effect sizes from the primary literature while improving statistical efficiency [3]. Following prior work, models were estimated with Restricted Maximum Likelihood (REML) and inference based on the t-distribution; given anticipated heterogeneity across studies, we adopted random-effects specifications.

Overall heterogeneity was examined using the Q statistic. Variance components for levels 1–3 were estimated using Cheung’s formulas, and one-sided likelihood-ratio tests were applied to the level-2 and level-3 variance components to determine their significance [5].

Standardized mean differences were expressed as Hedges’ g, with 95% confidence intervals (CI) and 95% prediction intervals (PI). Based on prior conventions, effect sizes were classified as: small (g < 0.20), small-to-moderate (g = 0.20–0.49), moderate (g = 0.50–0.79), and large (g ≥ 0.80).

To examine dose–response relationships between exercise and cognition, we conducted three-level meta-regressions with total intervention duration and weekly duration as predictors and standardized g as the outcome, specifying random intercepts at the study and within-study levels. Models were fitted via REML with t-tests for inference [3, 6]. Under the linear specification, we used predict() to obtain point estimates and 95% CIs; 95% PIs were computed from the sum of the mean prediction variance and the random-effects variance [7]. CI and PI lower bounds were obtained via linear interpolation and marked on figures. Additionally, we modeled potential nonlinearity using natural cubic splines, treating the linear model as a comparator; the joint significance of spline terms was evaluated with an F-test [8].

We also conducted an exploratory mediation analysis: three-level models were fit for the a-path (exercise → cognition), b-path (cognition → depressive symptoms), and c-path (exercise → depressive symptoms). Indirect effects (a × b) and the proportion mediated were estimated using the product-of-coefficients (Delta) method, with robustness checks based on cluster-robust variance estimation (CR1), Knapp–Hartung inference (with REML fitting), and maximum-likelihood z-tests to address small-sample and model-specification concerns.

Sensitivity and influence diagnostics proceeded as follows. Under a two-level random-effects model, we performed influence analysis and visualized multiple diagnostics—studentized residuals, DFFITS, Cook’s distance, leverage, and covariance ratios—to identify potentially influential effect sizes [6]. Under three-level random-effects models, we additionally implemented leave-one-study-out (LOSO) and leave-one-effect-out (LOEO) procedures, recording point estimates and 95% CIs and comparing their changes (Δβ) with the baseline model [9]. To account for dependence among multiple effects within studies, we applied CR2 cluster-robust variance corrections (via the clubSandwich package) and used Satterthwaite’s degrees-of-freedom approximation for tests and interval estimation [10]. If removing any single study or effect did not alter the direction or statistical significance of the pooled estimate, we considered the findings robust. (Within the mediation framework, we conducted LOSO and influence analyses only.)

Potential publication bias was assessed using funnel plots, Egger’s regression, and the trim-and-fill procedure under a two-level model. To address within-study dependence, we further implemented Egger-type regressions within a three-level random-effects framework. To explore whether funnel asymmetry might be attributable to study characteristics, we added prespecified correction factors (covariates) to the Egger model (not applied in the mediation analyses) and compared the magnitude and significance of the association between the standard error and effect size with and without these covariates.

**3.1 Study selection**

Table 3. Studies Excluded After Full-Text Screening (with Reasons)

| Reasons | Title |
| --- | --- |
| Acute exercise | Effects of Acute Exercise on Mood and Well-Being in Patients with Major Depressive Disorder |
|  | The Role of Depression in Short-Term Mood and Fatigue Responses to Acute Exercise |
|  | The impact of a single session of aerobic exercise on positive emotional reactivity in depression: Insight into individual differences from the late positive potential |
|  | Effects of Acute Exercise on Circulating Soluble Form of the Urokinase Receptor in Patients With Major Depressive Disorder |
|  | Acute Exercise Attenuates Negative Affect Following Repeated Sad Mood  Inductions in Persons Who Have Recovered From Depression |
|  | Influence of Exercise Intensity for Improving Depressed Mood in Depression: A Dose-Response Study |
|  | Dose-response effects of acute exercise intensity on state anxiety among women with depression |
|  | Magnitude, timing and duration of mood state and cognitive effects of acute  moderate exercise in major depressive disorder |
| No non-exercise control group | Dose-dependent changes in cognitive function with exercise augmentation for major depression: Results from the TREAD study |
| Non-target population | 有氧运动干预对癌症抑郁患者认知功能的影响研究 (Effect of aerobic exercise intervention on cognitive function of patients with cancer depression) |
|  | 广场舞对社区老年轻度认知障碍合并抑郁症状患者的干预效果 (The intervention effect of square dancing on elderly patients with mild cognitive impairment and depressive symptoms in the community) |
| Ineligible experimental intervention | Interacting with nature improves cognition and affect for individuals  with depression |
|  | Effects of cognitive behavioural therapy on verbal learning and  memory in major depression: Results of a randomized  controlled trial |

**3.2 Characteristics of included studies**

Table 4. Basic characteristics of the included studies

| Reference | Country | Age | Sample size/  Sex(F%) | Depression severity /Inpatient status | Outcome | Study design |
| --- | --- | --- | --- | --- | --- | --- |
| Brush et al.,2020 | USA | 20.23 | 66/74.24% | BDI-II:  E:22.06±8.03,  C:20.35±7.04/  Outpatients | Flankers task | RCT |
| Oertel-Knöchel et al.,2014 | Germany | 40.08 | 22/50% | BDI-II:  E:25.25±10.21,  C1:27.75±7.14,  C2:25.50±11.83/  Inpatients | Trail Making Test,  Symbol Coding,  Spatial Span,  Letter–number–span,  Hopkins Verbal Learning Test-Revised,  Brief Visuospatial Memory Test-Revised,  Animal naming | RCT |
| Brüchle et al.,2021 | Germany | 36.71 | 41/43.90% | BDI-II:  E:27.74±1.44,  C:26.11±1.77;  HAMD-17:  E:19.17±0.78,  C:17.83±0.75/  Inpatients | Trail making test,  Go-Nogo,  Tower of London, Stroop,  N-back,  Work performance series | Non-RCT |
| Bushert et al.,2019 | Germany | 47.37 | 30/63.33% | BDI-II:  E:22.7±8.53,  C:18.27±11.56;  HAMD-7:  E:11.00±3.42,  C:9.67±4.21/  Inpatients | Testbatteriezur Aufmerksamkeitsprüfung,  Digit span test,  Verbal learning and memory test,  Standard progressive matrices, Computergestütztes Kartensortierverfahren | RCT |
| Chan et al.,2012 | China | 46.25 | 33/81.6% | BDI-II:  E:28.29±14.84,  C:28.13±14.25;  HRSD:  E:12.06±4.48,  C:11.56±5.53/  Outpatients | Digit Vigilance Test | RCT |
| Foley.,2008 | New Zealand | 18-55 | 23/Unclear | BDI-II:  E:21.4±4.33,  C:20±7.08;  MADRAS:  E:28.80±9.86,  C:28.69±8.59/  Outpatients | Un-cued recall | RCT |
| Halappa et al.,2018 | India | 33.94 | 65/43.55% | HDRS:  E1:18.12±5,  E2:16.66±4.09,  C:18.17±4.15/  Outpatients | Digit span Test,  Trail Making Test,  Rey auditory verbal learning test | Non-RCT |
| Hoffman et al.,2008 | USA | 51.7 | 202/75.7% | HAMD:  Total:16.8±4.3/  Outpatients | Animal Naming，  Controlled Oral Word Association Test,  Digit Symbol Test,  Digit span Test,  Trail Making Test,  Ruff 2 & 7 Test,  Stroop,  Verbal Paired Associates Subtest from the Wechsler Memory Scale,  Logical Memory Subtest from the Wechsler Memory Scale | RCT |
| Imboden et al.,2020 | Switzerland | 39.9 | 42/47.6% | BDI:  E:27.4±9.1,  C:25.6±7.0;  HDRS17:  E:22.0±4.0,  C:20.9±2.6/  Inpatients | Testbatterie zur Aufmerksamkeitsprüfung,  Go-Nogo,  Working memory difficulty level 3,  Flexibility-verbal change | RCT |
| Khatri et al., 2001 | USA | 56.73 | 84/76.19% | BDI:  Total:21.55±7.54;  HAMD:  Total:18.04±3.63/  Outpatients | Logical Memory Subtest from the Wechsler Memory Scale,  Visual Reproduction Associates Subtest from the Wechsler Memory Scale,  Digit Symbol Test,  Digit span Test,  Trail Making Test,  Stroop | RCT |
| Krogh et al.,2009 | Denmark | 38.9 | 165/73.9% | BDI-II:  Total:31.0±8.1;  MADRS:  Total:22.1±5.3;  HRSD17:  Total:17.8±3.8  /Outpatients | Digit Span Test,  Subtracting Serial Sevens,  Trail making test,  Digit Symbol Test,  the S part of Verbal Fluency S and Animals,  the Buschke Test,  the Rey Complex Figure Test | RCT |
| Krogh et al.,2012 | Denmark | 41.55 | 115/66.96% | HRSD17:  E:19.2±4.7,  C:18.6±4.0;  BDI:  E:35.7±7.0,  C: 35.5±8.4  /Outpatients | the Buschke Test,  the Rey Complex Figure Test,  Digit Span Test,  Subtracting Serial Sevens,  Stroop,  Trail making test,  Digit Symbol Test,  the S part of Verbal Fluency S and Animals | RCT |
| Krogh.,2014 | Denmark | 41.35 | 79/67.09% | HRSD17:  E:19±3.9,  C:18.9±4.6;  /Outpatients | the Buschke Test,  the Rey Complex Figure Test | RCT |
| Lavretsky et al.2011 | USA | 70.55 | 73/62.00% | HDRS:  E:8.2±5.5，  C:9.8±5.8/  /Outpatients | the California Verbal Learning Test II,  Trail making test,  Mini-Mental State Examination | RCT |
| Lavretsky et al.,2022 | USA | 69.3 | 178/72.47% | HAMD:  E:19.0±4.0，  C:19.3±3.9/  /Outpatients | the California Verbal Learning Test II,  Trail making test,  Stroop,  Controlled Oral Word Association test,  Animal Fluency,  Boston Naming Test | RCT |
| Leleikiene et al.,2018 | Lithuania | 54.6 | 52/Unclear | Moderate /  Outpatients | Go-Nogo,  the matching grids test,  the mathematical processing test,  the 2-choice reaction time test, the simple reaction time test. | RCT |
| Olson et al.2017 | USA | 21.1 | 30/80.00% | BDI-II:  E:24.5±11.5,  C:24.3±11.9/  Outpatients | Flanker Task | RCT |
| Schwefel et al.,2025 | Germany | 37.3 | 86/45.35% | HAMD:  E1:13.2±3.9  E2:13.1±3.4  C:12.7±4.1  BDI-II  E1:26.0±8.6  E2:28.1±6.3  C:26.8±7.6/  Outpatients | N-back | RCT |
| Sharma et al.,2006 | India | 31.77 | 30/36.67% | HAMD:  E:21.27±4.35  C:19.47±3.98/  Outpatients | Letter Cancellation Test,  Trail Making Test,  Ruff Figural Fluency Test,  Digit Span Test | RCT |
| Yun et al.,2025 | China | 19.74 | 41/63.46% | BDI-II:  E:18.4±5.82  C:17.62±3.10/  Outpatients | Go-Nogo | RCT |
| Chen et al.,2021 | China | 31.5 | 125/66.40% | HAMD:  E:10.3±3.4,  C:10.0±3.8/  Outpatients | Digit Span Test,  Trail making test,  Continuous performance task, Digit Span,  Verbal Fluency test | RCT |
| Zhang et al.,2019 | China | 31.8 | 125/66.40% | HAMD:  E:10.3±3.4,  C:10.0±3.8/  Outpatients | Digit Span Test,  Trail making test,  Continuous performance task, Digit Span,  Verbal Fluency test | RCT |
| Fu et al.,2022 | China | 14.87 | 90/54.44% | HAMD:  E:27.22±4.52,  C:26.93±4.71/  Inpatients | Digit Span Test,  Trail making test | RCT |
| Fu et al.,2024 | China | 12-18 | 30/Unclear | HAMD: E:27.1±5.7, C:29.0±3.4/  Inpatients | the Wisconsin Card Sorting Test | Non-RCT |
| Shen et al.,2023 | China | 44.9 | 78/58.94% | HAMD17: E:22.5±4.0，  C:21.9±3.6/ Outpatients | Repeatable Battery for the  Assessment of Neuropsychological Status | RCT |
| Zhang et al.,2022 | China | 50.59 | 39/89.74 | BDI-II: Total:23.32±1.06/  Outpatients | Stroop,  Tower of London,  the Wisconsin Card Sorting Test | RCT |
| Liu et al.,2022 | China | 40.08 | 148/50.00% | HAMD: E1:28.61±5.08, E2:29.44±4.98, C:29.33±4.75/ Inpatients | the Montreal Cognitive Assessment | RCT |
| Zheng et al.,2019 | China | 36.34 | 60/55.00% | MADRS: E:26.60±3.44,  C:26.80±6.03/ Inpatients | Repeatable Battery for the  Assessment of Neuropsychological Status | RCT |
| Vollbehr et al.,2023 | Netherlands | 25.05 | 171/100% | HDRS:  E:19.05±5.81,  C:18.07±6.06/  Outpatients | Exogenous Cueing Task | RCT |
| Kumari et al.,2023 | India | 18-45 | 64/37.50% | Mild-to-moderate/  Inpatients | Digit Span Test | RCT |
| Luttenberger et al.,2015 | Germany | 43.91 | 47/57.50% | Moderate/  Outpatients | D2-R | RCT |

RCT for randomized controlled trail. BDI for Beck Depression Inventory.BDI-II for Beck Depression Inventory-II. HAMD/HDRS/HRSD for Hamilton Depression Rating Scale. MADRS for Montgomery-Asberg Depression Rating Scale. E for experimental group. C for comparison group.1 for first group. 2 for second group. Age is presented as reported in the original studies. Some studies provided mean ± SD, while others only reported age ranges.

Table 5. Intervention characteristics of the included studies

| Reference | Intervention content | Control group |
| --- | --- | --- |
| Brush et al.,2020 | Type:  E: Arobic exercise (Treadmill or Cycle)  Intensity: Moderate (40–65%HR reserve)  Frequency: 3 days/week  Duration: 45 min  Cycle: 8 weeks | Light stretching |
| Oertel-Knöchel et al.,2014 | Type:  E: Arobic exercise (Aerobic with boxing and circuit training) combined with cognitive training  Intensity: Moderate ( 60-70%HRmax)  Frequency: 3 days/week  Duration: 45 min  Cycle: 4 weeks | Relaxation training/Waiting combined with cognitive training |
| Brüchle et al.,2021 | Type: Multi-component exercise (Coordination,  Endurance or Strength training) combined with usual care  Intensity: Moderate  Frequency: 3 days/week  Duration: 60 min  Cycle: 3 weeks | The cognitive game combined with usual care |
| Bushert et al.,2019 | Type:  E: Arobic exercise ( Walking, Nordic walking, or Running) combined with drug therapy  Intensity: Moderate-high (85%HRmax)  Frequency: 2-3 days/week  Duration: 30 min  Cycle: 3-4 weeks | occupational or art therapy combined with drug therapy |
| Chan et al.,2012 | Type:  E: Mind–Body exercises (Shidejian) combined with drug therapy  Intensity: Low  Frequency: 1 day/week  Duration: 90 min  Cycle: 10 weeks | Waitlist combined with drug therapy |
| Foley et al.,2008 | Type:  E: Aerobic exercise  Intensity: Moderate  Frequency: 3 days/week  Duration: 30-40 min  Cycle: 10 weeks | Stretching |
| Halappa et al.,2018 | Type:  E1: Mind–Body exercises (Yoga) combined with antidepressant medication  E2: Mind–Body exercises (Yoga)  Intensity: Low  Frequency: 2 days/week  Duration: 60 min  Cycle: 12 weeks | Antidepressant medication |
| Hoffman et al.,2008 | Type:  E1: Aerobic exercise  Intensity:Moderate-high (70-85%HR reserve)  Frequency: 3 days/week  Duration: 45 min  Cycle: 16 weeks | Placebo pill or sertraline |
| Imboden et al.,2020 | Type: E1:Aerobic exercise (Bicycles) combined with standard inpatient treatment  Intensity:Moderate(60-75%HRmax)  Frequency: 3days/week  Duration: 45min  Cycle: 6 weeks | Light stretching combined with standard inpatient treatment |
| Khatri et al., 2001 | Type:  E: Aerobic exercise (Cycle, Walking or Jogging)  Intensity: Moderate-high (70-85%HR reserve)  Frequency: 3 days/week  Duration: 45 min  Cycle: 16 weeks | Antidepressant medication |
| Krogh et al.,2009 | Type:  E1: Aerobic exercise (Cycle,Walking or Jogging),  E2: Strength training  Intensity:  E1: Moderate-high (70-89%HR max),  E2: Moderate-high (50-75%RM)  Frequency: 2 days/week  Duration: 90 min  Cycle: 16 weeks | Light stretching |
| Krogh et al.,2012 | Type:  E1: Aerobic exercise (Stationary cycle ergometer)  Intensity: Moderate-high (65-80%VO_2_max)  Frequency: 3 days/week  Duration: 45 min  Cycle: 12 weeks | Light stretching |
| Krogh et al.,2014 | Type: Aerobic exercise (Stationary cycle ergometer)  Intensity: Moderate-high (80%HRmax)  Frequency: 3 days/week  Duration: 45 min  Cycle: 12 weeks | Light stretching |
| Lavretsky et al.2011 | Type:  E: Mind–Body exercises (Taichi) combined with drug therapy  Intensity: Low  Frequency: 1 day/week  Duration: 120 min  Cycle: 10 weeks | Drug therapy combined with health education |
| Lavretsky et al.2022 | Type:  E: Mind–Body exercises (Taichi) combined with drug therapy  Intensity: Low  Frequency: 1 days/week  Duration: 60 min  Cycle: 12 weeks | Drug therapy combined with health education |
| Leleikiene et al.,2018 | Type:  E1:Aerobic exercise combined with drug therapy  E2: Strength exercises combined with drug therapy  Intensity: Moderate  Frequency: 3days/week  Duration: 45min  Cycle: 4 weeks | Drug therapy |
| Olson et al.2017 | Type:  E: Aerobic exercise (Treadmill or Cycle) combined with drug therapy,  Intensity: Moderate (40-65% HR reserve)  Frequency: 3 days/week  Duration: 30-45 min  Cycle: 8 weeks | Light stretching |
| Schwefel et al.,2025 | Type:  E1:Aerobic exercise (Bicycle ergometer, Running or Nordic walking, and aerobic body workout )  E2:Aerobic exercise (cycling, walking and stretching)  Intensity:  E1:Moderate-high (55-85% HR reserve)  E2:Low (20-30% HR reserve)  Frequency: 2 days/week  Duration: 60 min  Cycle: 12 weeks | Waitlist |
| Sharma et al.,2006 | Type:  E: Mind–Body exercises (Yoga) combined with conventional antidepressant treatment  Intensity: Low  Frequency: 3 days/week  Duration: 30 min  Cycle: 8 weeks | Conventional antidepressant treatment |
| Yun et al.,2025 | Type:  E: Multi-component exercise (Taekwondo)  Intensity: Moderate (64-76%HRmax)  Frequency: 3 days/week  Duration: 30 min  Cycle: 8 weeks | Sit quietly |
| Chen et al.,2021 | Type:  E:Aerobic exercise (Jogging or Ball) combined with antidepressant medication  Intensity:Moderate (64-76% HRmax)  Frequency: 3 days/week  Duration: 30-60min  Cycle: 16 weeks | Antidepressant medication |
| Zhang et al.,2019 | Type:  E: Aerobic exercise (Jogging) combined with antidepressant medication  Intensity: Moderate (170-age)  Frequency: 3 days/week  Duration: 30 min  Cycle: 8 weeks | Antidepressant medication |
| Fu et al.,2022 | Type:  E: Aerobic exercise (Jogging) combined with  antidepressant medication  Intensity: Moderate-high (60-80%HRmax)  Frequency: 5 days/week  Duration: 40-50 min  Cycle: 8 weeks | Antidepressant medication |
| Fu et al.,2024 | Type:  E: Aerobic exercise (Cycle) combined with antidepressant medication  Intensity: Moderate (60-69%HRmax)  Frequency: 4 days/week  Duration: 30 min  Cycle: 4 weeks | Antidepressant medication |
| Shen et al.,2023 | Type:  E: Mind–Body exercises (Yoga) combined with conventional rehabilitation  Intensity: Low  Frequency: 2 days/week  Duration: 90 min  Cycle: 8 weeks | Conventional rehabilitation |
| Zhang et al.,2022 | Type:  E: Mind–Body exercises (Taichi)  Intensity: Low  Frequency: 2 days/week  Duration: 90 min  Cycle: 12 weeks | Waitlist |
| Liu et al.,2022 | Type:  E1: Aerobic exercise (Bicycle, Running or Nordic walking, and Aerobic body workout ) combined with conventional rehabilitation  E2: Strength exercises combined with conventional rehabilitation  Intensity: Moderate-high (60-85% HRmax)  Frequency: 3 days/week  Duration: 30-60 min  Cycle: 12 weeks | Conventional rehabilitation |
| Zheng et al.,2019 | Type:  E: Aerobic exercise (Bicycle) combined with conventional rehabilitation  Intensity: Moderate (60% HRmax)  Frequency: 5 days/week  Duration: 30 min  Cycle: 4 weeks | Conventional rehabilitation |
| Vollbehr et al.,2023 | Type:  E: Mind–Body exercises (Yoga) combined with standard care  Intensity: Low  Frequency: 5 days/week  Duration: 90 min  Cycle: 9 weeks | Standard care |
| Kumari et al.,2023 | Type:  E: Mind–Body exercises (Yoga) combined with conventional antidepressant medication  Intensity: Low  Frequency: 5 days/week  Duration: 60 min  Cycle: 12 weeks | Conventional antidepressant  medication |
| Luttenberger et al.,2015 | Type:  E: Mind–Body exercises (Bouldering)  Intensity: Low  Frequency: 1 day/week  Duration: 180 min  Cycle: 8 weeks | Waitlist |

E for experimental group. C for comparison group.1 for first group. 2 for second group. HRmax Maximum Heart Rate. HR reserve for Heart Rate Reserve. VO₂max for Maximal oxygen uptake.

RM for Repetition Maximum

**3.4 Meta-analytic results**


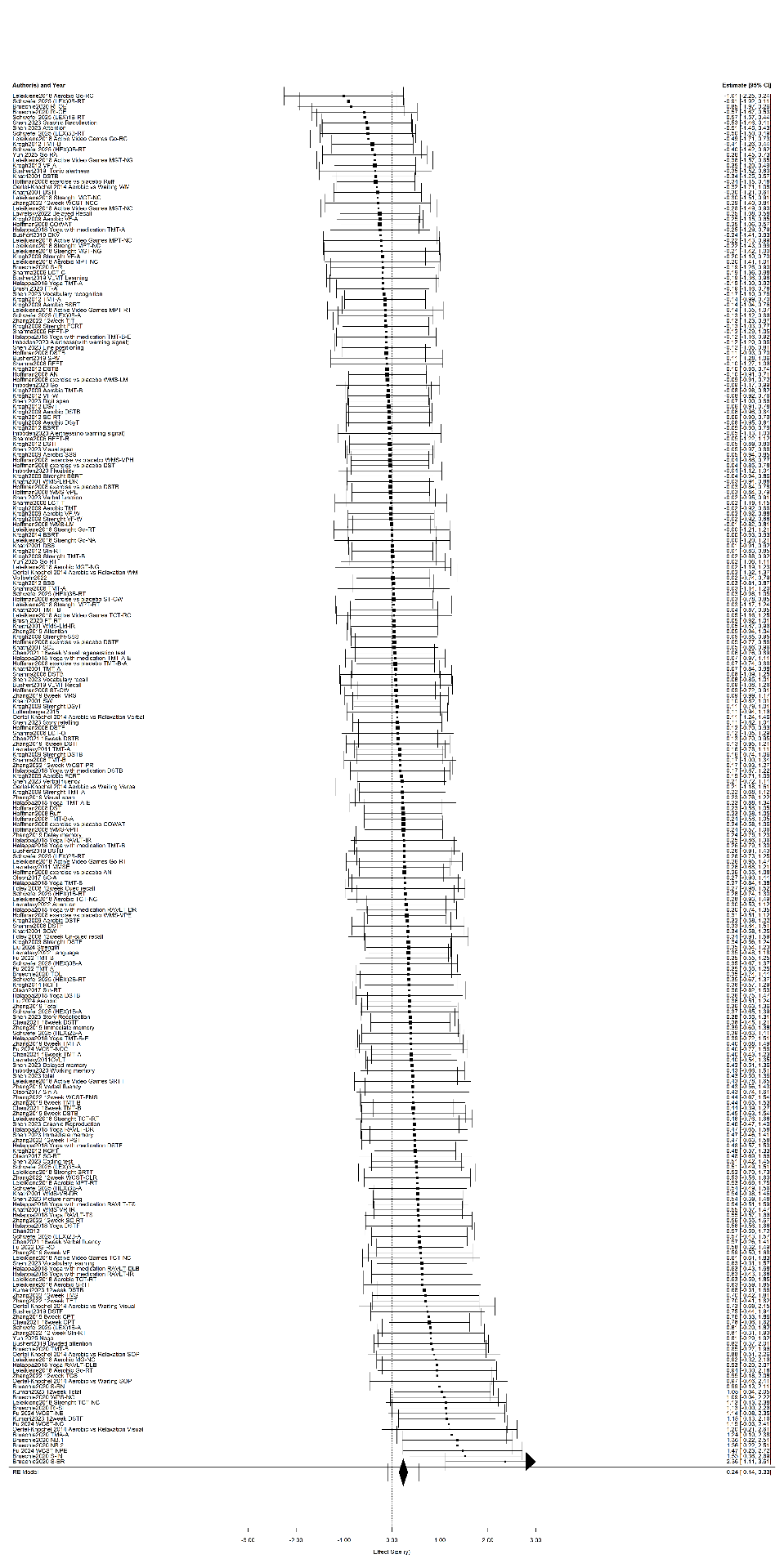


Figure 1. Forest plot of the effects of exercise on cognitive function in patients with depression.

**3.4.2 Moderator analyses**

Table 5. Subgroup analyses

| Moderators | k | n | g | 95%CI | P | Test of  Moderators | Level2 | Level3 | GRADE |
| --- | --- | --- | --- | --- | --- | --- | --- | --- | --- |
| Cognitive subdomain |  |  |  |  |  | F(5,250)=0.25,  P=0.940 | 2.11% | 9.01% |  |
| Executive function | 22 | 1560 | 0.22 | 0.10,0.34 | ＜0.001 |  |  |  | High |
| Memory | 16 | 1407 | 0.29 | 0.13,0.45 | ＜0.001 |  |  |  | High |
| Attention | 15 | 1211 | 0.23 | 0.03,0.43 | 0.024 |  |  |  | Moderate |
| Verbal fluency | 8 | 967 | 0.20 | -0.04,0.43 | 0.103 |  |  |  | Very low |
| Processing speed | 12 | 1111 | 0.19 | -0.02,0.41 | 0.073 |  |  |  | Very low |
| Over | 4 | 311 | 0.37 | -0.08,0.81 | 0.105 |  |  |  | Low |
| Exercise type |  |  |  |  |  | F(3,252)=0.51,P=0.675 | 2.12% | 6.95% |  |
| Aerobic exercise | 19 | 1395 | 0.20 | 0.09,0.31 | ＜0.001 |  |  |  | High |
| Strength exercise | 3 | 212 | 0.19 | -0.08,0.46 | 0.167 |  |  |  | Very low |
| Mind-body exercise | 10 | 725 | 0.27 | 0.11,0.44 | 0.001 |  |  |  | Moderate |
| Multicomponent exercise | 3 | 108 | 0.36 | 0.08,0.64 | 0.012 |  |  |  | Very low |
| Session duration |  |  |  |  |  | F(1,254)=0.32,P=0.570 | 2.43% | 9.37% |  |
| 30-60 min | 24 | 1697 | 0.25 | 0.15,0.36 | ＜0.001 |  |  |  | High |
| ＞60 min | 7 | 578 | 0.19 | -0.02,0.39 | 0.078 |  |  |  | Low |
| Exercise intensity |  |  |  |  |  | F(2,253)=5.05,P=0.007 | 2.61% | 2.29% |  |
| Low | 11 | 779 | 0.23 | 0.10,0.36 | ＜0.001 |  |  |  | Moderate |
| Moderate | 12 | 659 | 0.36 | 0.23,0.50 | ＜0.001 |  |  |  | High |
| Moderate to high | 9 | 891 | 0.08 | -0.03,0.20 | 0.149 |  |  |  | Low |
| Exercise frequency |  |  |  |  |  | F(1,254)=0.70,P=0.402 | 1.44% | 9.48% |  |
| 1-2 days/week | 11 | 852 | 0.19 | 0.03,0.34 | 0.018 |  |  |  | High |
| 3-5 days/week | 20 | 1423 | 0.27 | 0.15,0.39 | ＜0.001 |  |  |  | High |
| Exercise Cycle |  |  |  |  |  | F(2,253)=1.95,P=0.144 | 2.21% | 7.19% |  |
| 3-6 weeks | 8 | 379 | 0.37 | 0.19,0.54 | ＜0.001 |  |  |  | Moderate |
| 8-12 weeks | 19 | 1348 | 0.22 | 0.10,0.35 | ＜0.001 |  |  |  | High |
| ＞12 weeks | 4 | 548 | 0.11 | -0.07,0.30 | 0.225 |  |  |  | Low |
| Inpatient status |  |  |  |  |  | F(1,254)=6.71,P=0.010 | 2.66% | 6.00% |  |
| Inpatient | 9 | 473 | 0.42 | 0.25,0.59 | ＜0.001 |  |  |  | Moderate |
| Outpatient | 22 | 1802 | 0.17 | 0.07,0.26 | 0.001 |  |  |  | High |
| Age |  |  |  |  |  | F(3,252)=1.58,P=0.195 | 2.12% | 8.14% |  |
| Adolescents | 2 | 120 | 0.69 | 0.24,1.14 | 0.003 |  |  |  | Very low |
| Young adults | 17 | 1269 | 0.25 | 0.12,0.37 | ＜0.001 |  |  |  | Low |
| Middle adults | 10 | 688 | 0.17 | 0.02,0.33 | 0.028 |  |  |  | High |
| Older adults | 2 | 198 | 0.20 | -0.22,0.61 | 0.360 |  |  |  | Vey low |
| Study design |  |  |  |  |  | F(1,254)=10.74,P=0.001 | 1.47% | 3.84% |  |
| RCT | 28 | 2139 | 0.17 | 0.09,0.26 | ＜0.001 |  |  |  | High |
| Non-RCT | 3 | 136 | 0.57 | 0.35,0.80 | ＜0.001 |  |  |  | Very low |
| Intervention Content |  |  |  |  |  | F(1,254)=5.23,P=0.023 | 2.87% | 4.93% |  |
| Exercise only | 13 | 956 | 0.13 | 0.01,0.24 | 0.037 |  |  |  | High |
| Exercise + Other | 19 | 1342 | 0.31 | 0.20,0.42 | ＜0.001 |  |  |  | High |
| Control group |  |  |  |  |  | F(1,254)=0.05,P=0.823 | 2.50% | 9.20% |  |
| Active control | 26 | 2004 | 0.24 | 0.14,0.34 | ＜0.001 |  |  |  | High |
| Passive Control | 7 | 383 | 0.22 | 0.02,0.42 | 0.031 |  |  |  | High |
| Language |  |  |  |  |  | F(1,254)=1.73,P=0.189 | 2.11% | 8.75% |  |
| Chinese | 6 | 483 | 0.36 | 0.15,0.57 | ＜0.001 |  |  |  | Moderate |
| English | 25 | 1792 | 0.21 | 0.10,0.31 | ＜0.001 |  |  |  | moderate |

K for Number of included studies. N for Total number of participants

**3.5 Sensitivity and influence analyses**


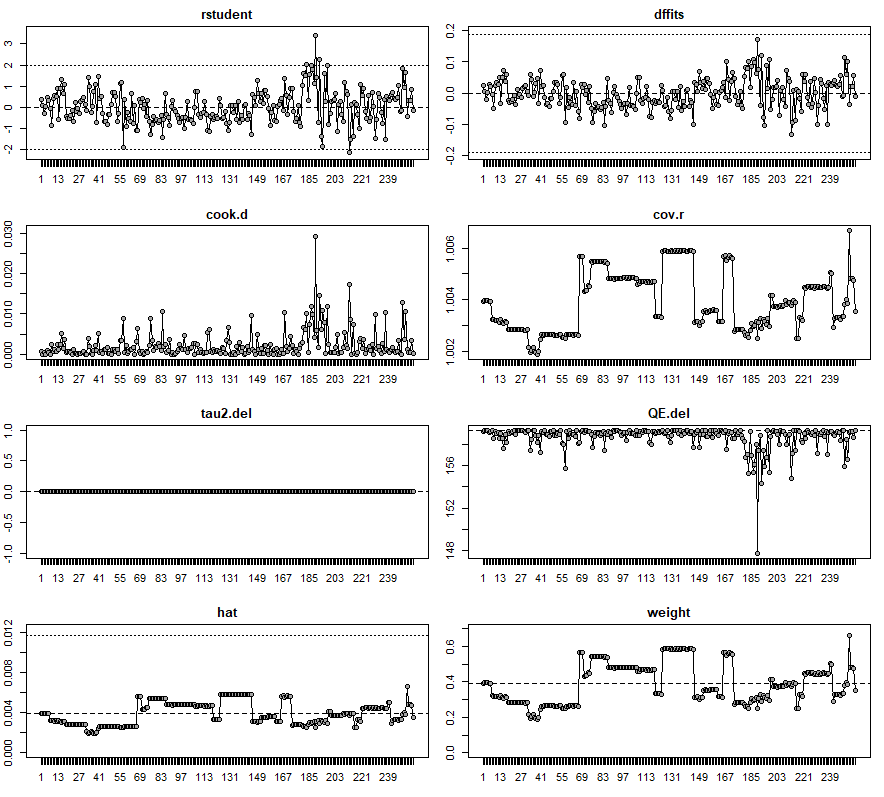


Figure 2. Influence analysis of the primary model for the effects of exercise on cognitive function in patients with depression.

**3.6 Publication bias**


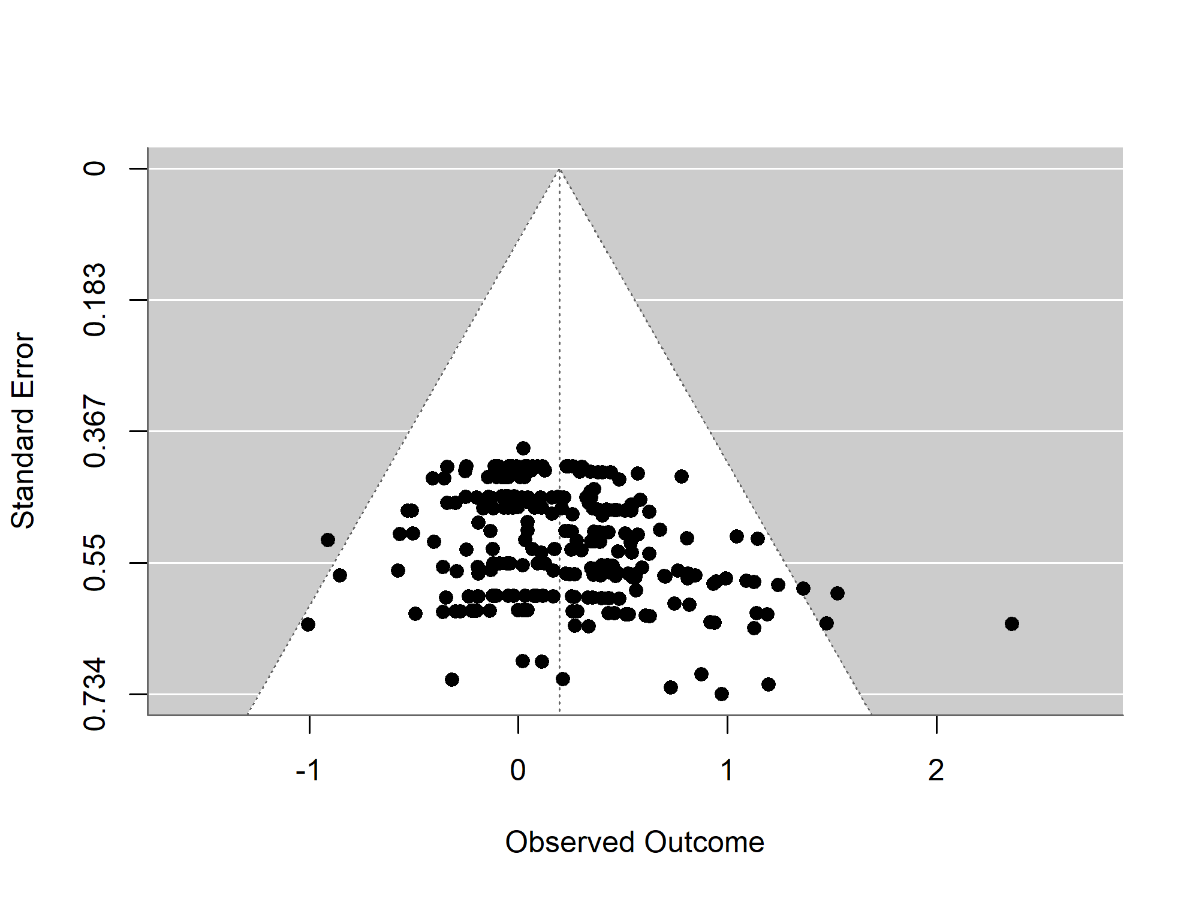


Figure 3. Funnel plot of the intervention effects of exercise on cognitive function in patients with depression.
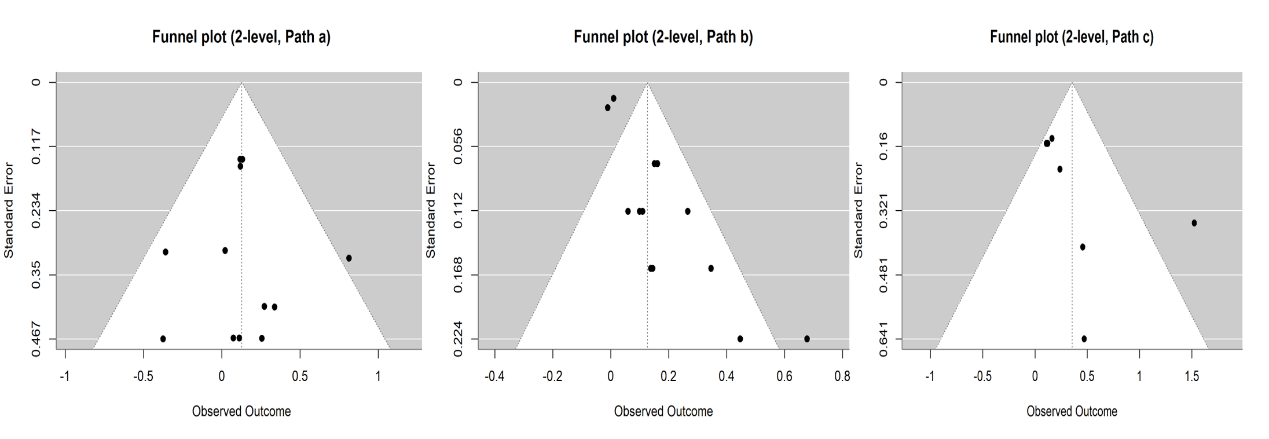


Figure 4. Funnel plot for the mediation analysis.

**3.7 Certainty of evidence**

Table 6. GRADE certainty of evidence assessment.

| Moderators | k | n | g | 95%CI | Study limitations | Consistency | Directness | Precision | Publication bias | Evidence Quality* |
| --- | --- | --- | --- | --- | --- | --- | --- | --- | --- | --- |
| Cognitive function | 31 | 2434 | 0.24 | 0.14,0.33 | Downgrade 1 Level^1^ | None | None | None | None | Moderate |
| Cognitive subdomain |  |  |  |  |  |  |  |  |  |  |
| Executive function | 22 | 1560 | 0.22 | 0.10,0.34 | Downgrade 1 Level^1^ | None | None | None | None | Moderate |
| Memory | 16 | 1407 | 0.29 | 0.13,0.45 | Downgrade 1 Level^1^ | None | None | None | None | Moderate |
| Attention | 15 | 1211 | 0.23 | 0.03,0.43 | Downgrade 1 Level^1^ | None | None | None | Downgrade 1 Level^2^ | Low |
| Verbal fluency | 8 | 967 | 0.20 | -0.04,0.43 | Downgrade 1 Level^1^ | None | None | Downgrade 1 Leve1^3^ | Downgrade 1 Level^2^ | Very low |
| Processing speed | 12 | 1111 | 0.19 | -0.02,0.41 | Downgrade 1 Level^1^ | None | None | Downgrade 1 Leve1^3^ | None | Low |
| Over | 4 | 311 | 0.37 | -0.08,0.81 | Downgrade 1 Level^1^ | None | None | Downgrade 2 Level^4^ | Downgrade 1 Level^2^ | Very low |
| Exercise type |  |  |  |  |  |  |  |  |  |  |
| Aerobic exercise | 19 | 1395 | 0.20 | 0.09,0.31 | Downgrade 1 Level^1^ | None | None | None | None | Moderate |
| Strength exercise | 3 | 212 | 0.19 | -0.08,0.46 | Downgrade 1 Level^1^ | None | None | Downgrade 2 Level^4^ | Downgrade 1 Level^2^ | Very low |
| Mind-body exercise | 10 | 725 | 0.27 | 0.11,0.44 | Downgrade 1 Level^1^ | None | None | None | None | Moderate |
| Multicomponent exercise | 3 | 108 | 0.36 | 0.08,0.64 | Downgrade 1 Level^1^ | None | None | Downgrade 2 Level^4^ | Downgrade 1 Level^2^ | Very low |
| Session duration |  |  |  |  |  |  |  |  |  |  |
| 30-60 min | 24 | 1697 | 0.25 | 0.15,0.36 | Downgrade 1 Level^1^ | None | None | None | None | Moderate |
| ＞60 min | 7 | 578 | 0.19 | -0.02,0.39 | Downgrade 1 Level^1^ | None | None | Downgrade 2 Level^4^ | None | Very low |
| Exercise intensity |  |  |  |  |  |  |  |  |  |  |
| Low | 11 | 779 | 0.23 | 0.10,0.36 | Downgrade 1 Level^1^ | None | None | None | None | Moderate |
| Moderate | 12 | 659 | 0.36 | 0.23,0.50 | Downgrade 1 Level^1^ | None | None | None | None | Moderate |
| Moderate to high | 9 | 891 | 0.08 | -0.03,0.20 | Downgrade 1 Level^1^ | None | None | Downgrade 1 Leve1^3^ | Downgrade 1 Level^2^ | Very Low |
| Exercise frequency |  |  |  |  |  |  |  |  |  |  |
| 1-2 days/week | 11 | 852 | 0.19 | 0.03,0.34 | Downgrade 1 Level^1^ | None | None | None | None | Moderate |
| 3-5 days/week | 20 | 1423 | 0.27 | 0.15,0.39 | Downgrade 1 Level^1^ | None | None | None | None | Moderate |
| Exercise Cycle |  |  |  |  |  |  |  |  |  |  |
| 3-6 weeks | 8 | 379 | 0.37 | 0.19,0.54 | Downgrade 1 Level^1^ | None | None | Downgrade 1 Leve1^5^ | None | Low |
| 8-12 weeks | 19 | 1348 | 0.22 | 0.10,0.35 | Downgrade 1 Level^1^ | None | None | None | None | Moderate |
| ＞12 weeks | 4 | 548 | 0.11 | -0.07,0.30 | Downgrade 1 Level^1^ | None | None | Downgrade 1 Leve1^3^ | Downgrade 1 Level^2^ | Very low |
| Inpatient status |  |  |  |  |  |  |  |  |  |  |
| Inpatient | 9 | 473 | 0.42 | 0.25,0.59 | Downgrade 1 Level^1^ | None | None | Downgrade 1 Leve1^5^ | None | Low |
| Outpatient | 22 | 1802 | 0.17 | 0.07,0.26 | Downgrade 1 Level^1^ | None | None | None | None | Moderate |
| Age |  |  |  |  |  |  |  |  |  |  |
| Adolescents | 2 | 120 | 0.69 | 0.24,1.14 | Downgrade 1 Level^1^ | None | None | Downgrade 2 Level^4^ | Downgrade 1 Level^2^ | Very low |
| Young adults | 17 | 1269 | 0.25 | 0.12,0.37 | Downgrade 1 Level^1^ | None | None | None | Downgrade 1 Level^2^ | Low |
| Middle adults | 10 | 688 | 0.17 | 0.02,0.33 | Downgrade 1 Level^1^ | None | None | None | None | Moderate |
| Older adults | 2 | 198 | 0.20 | -0.22,0.61 | Downgrade 1 Level^1^ | None | None | Downgrade 2 Level^4^ | Downgrade 1 Level^2^ | Very low |
| Study design |  |  |  |  |  |  |  |  |  |  |
| RCT | 28 | 2139 | 0.17 | 0.09,0.26 | Downgrade 1 Level^1^ | None | None | None | None | Moderate |
| Non-RCT | 3 | 136 | 0.57 | 0.35,0.80 | Downgrade 1 Level^1^ | None | None | Downgrade 2 Level^4^ | Downgrade 1 Level^2^ | Very low |
| Intervention condition |  |  |  |  |  |  |  |  |  |  |
| Exercise only | 13 | 956 | 0.13 | 0.01,0.24 | Downgrade 1 Level^1^ | None | None | None | None | Moderate |
| Exercise + Other | 19 | 1342 | 0.31 | 0.20,0.42 | Downgrade 1 Level^1^ | None | None | None | None | Moderate |
| Control group |  |  |  |  |  |  |  |  |  |  |
| Active control | 26 | 2004 | 0.24 | 0.14,0.34 | Downgrade 1 Level^1^ | None | None | None | None | Moderate |
| Passive Control | 7 | 383 | 0.22 | 0.02,0.42 | Downgrade 1 Level^1^ | None | None | None | None | Moderate |
| Language |  |  |  |  |  |  |  |  |  |  |
| Chinese | 6 | 483 | 0.36 | 0.15,0.57 | Downgrade 1 Level^1^ | None | None | Downgrade 1 Leve1^5^ | None | Low |
| English | 25 | 1792 | 0.21 | 0.10,0.31 | Downgrade 1 Level^1^ | None | None | None | Downgrade 1 Level^2^ | Low |
| Mediation analysis |  |  |  |  |  |  |  |  |  |  |
| Path A | 5 | 521 | 0.13 | -0.01,0.26 | Downgrade 1 Level^1^ | None | None | Downgrade 1 Leve1^5^ | None | Low |
| Path B | 5 | 521 | 0.15 | 0.07,0.23 | Downgrade 1 Level^1^ | None | None | Downgrade 1 Leve1^5^ | Downgrade 1 Level^2^ | Very low |
| Path C | 5 | 521 | 0.36 | 0.09,0.69 | Downgrade 1 Level^1^ | None | None | Downgrade 1 Leve1^5^ | None | Low |

1 for insufficient reporting of randomization/allocation concealment, with few studies at overall low risk of bias; 2 for downgraded by one level for publication bias; 3 for the 95% confidence interval for this subgroup crosses zero and spans the prespecified minimally important difference threshold (+0.2 SD); 4 for the 95% confidence interval for this subgroup crosses zero and spans the prespecified minimally important difference threshold (+0.2 SD), in addition, small sample size; 5 for small sample size.

**References**

1. Lehrer EJ, Wang M, Sun Y, Zaorsky NG. An Introduction to Meta-Analysis. International journal of radiation oncology, biology, physics. 2023 Mar 1;115(3):564-71.

2. Cumpston M, Li T, Page MJ, Chandler J, Welch VA, Higgins JP, et al. Updated guidance for trusted systematic reviews: a new edition of the Cochrane Handbook for Systematic Reviews of Interventions. The Cochrane database of systematic reviews. 2019 Oct 3;10(10):Ed000142.

3. Assink M, Wibbelink CJ. Fitting three-level meta-analytic models in R: A step-by-step tutorial. The Quantitative Methods for Psychology. 2016;12(3):154-74.

4. Cui X, Zhang S, Yu S, Ding Q, Li X. Does working memory training improve emotion regulation and reduce internalizing symptoms? A pair of three-level meta-analyses. Behaviour research and therapy. 2024 Aug;179:104549.

5. Cheung MW. Modeling dependent effect sizes with three-level meta-analyses: a structural equation modeling approach. Psychological methods. 2014 Jun;19(2):211-29.

6. Viechtbauer W. Conducting meta-analyses in R with the metafor package. Journal of statistical software. 2010;36(3):1-48.

7. Riley RD, Higgins JP, Deeks JJ. Interpretation of random effects meta-analyses. BMJ (Clinical research ed). 2011 Feb 10;342:d549.

8. Orsini N, Li R, Wolk A, Khudyakov P, Spiegelman D. Meta-analysis for linear and nonlinear dose-response relations: examples, an evaluation of approximations, and software. American journal of epidemiology. 2012 Jan 1;175(1):66-73.

9. Hedges LV, Tipton E, Johnson MC. Robust variance estimation in meta-regression with dependent effect size estimates. Research synthesis methods. 2010 Jan;1(1):39-65.

10. Tipton E. Small sample adjustments for robust variance estimation with meta-regression. Psychological methods. 2015 Sep;20(3):375-93.
